# Transcriptome profile analysis reveals the regulation mechanism of floral sex differentiation in *Jatropha curcas* L

**DOI:** 10.1038/s41598-017-16545-5

**Published:** 2017-11-27

**Authors:** Wenkai Hui, Yuantong Yang, Guojiang Wu, Changcao Peng, Xiaoyang Chen, Mohamed Zaky Zayed

**Affiliations:** 1 National Engineering Laboratory for Forest Tree Breeding, College of Biological Science and Technology, Beijing Forestry University, Beijing, 100083 P.R. China; 2 State Key Laboratory for Conservation and Utilization of Subtropical Agro-bioresources, Guangdong Key Laboratory for Innovative Development and Utilization of Forest Plant Germplasm, College of Forestry and Landscape Architecture, South China Agricultural University, Guangzhou, 510642 P.R. China; 3 Key Laboratory of Plant Resources Conservation and Sustainable Utilization, South China Botanical Garden, Chinese Academy of Sciences, Guangzhou, 510650 P.R. China; 4 Forestry and Wood Technology Department, Faculty of Agriculture (EL-Shatby), Alexandria University, Alexandria, Egypt

## Abstract

The seeds of *Jatropha curcas* contain a high percentage of biodiesel. However, low seed yield which was limited by its poor female flowers was a bottleneck for its utilization. Here, we compared the transcriptomic profiles of five different samples during floral sex differentiation stages using Illumina Hiseq 4000. Our results showed that hundreds of differentially expressed genes (DEGs) were detected in floral sex initiation period, but thousands of DEGs were involved in the stamens and ovules development process. Moreover, the DEGs were mainly shown up-regulation in male floral initiation, but mainly down-regulation in female floral initiation. Male floral initiation was associated with the flavonoid biosynthesis pathway while female floral initiation was related to the phytohormone signal transduction pathway. Cytokinin (CTK) signaling triggered the initiation of female floral primordium, thereafter other phytohormones co-promoted the female floral development. In addition, the floral organ identity genes played important roles in floral sex differentiation process and displayed a general conservation of the ABCDE model in *J. curcas*. To the best of our knowledge, this data is the first comprehensive analysis of the underlying regulatory mechanism and the related genes during floral sex differentiation in *J. curcas*, which help in engineering high-yielding varieties of *J. curcas*.

## Introduction

*Jatropha curcas* L., commonly known as the physic nut and belongs to the family Euphorbiaceae. It is a native species of Mexico and Central America, is widely distributed in the tropical and subtropical regions^1^. *J. curcas* is a bioenergy tree and its seeds contain a high oil content, which has the potential to be exploited as a source of biofuel^2^. However, the low seed yield of *J. curcas* is a major drawback in its utilization as bioenergy plant, which was caused by the lower number of female flowers. Therefore, revealing the potential regulatory mechanism involved in floral sex differentiation in *J. curcas*, especially female floral differentiation, is critical for the improvement of female flowers and the cultivation of high-yielding *J. curcas* germplasms.

Angiosperms mostly consist of bisexual flowers and but also bear unisexual flowers, which may be monoecious or dioecious^3^. To understand the potential regulatory events during floral sex differentiation, it is important to understand the different pathways used by the plant for its propagation. The common floral organs are initiated in the whole floral primordium, but the specific differentiation of stamens or pistils governs the formation of unisexual flowers^4^. In *J. curcas*, the differentiation of the floral primordium to form male and female flowers is regulated by some endogenous induction effects^5^, which may affect the action of homeotic genes to initiate the formation of gynoecium or androecium meristem. The availability of the *J. curcas* reference genome assembly has facilitated further fundamental and applied studies on this plant^6,7^. Comparative transcriptome analysis between gynoecious and monoecious *J. curcas* plants revealed 32 genes related to the floral development and 70 involved in phytohormone biosynthesis and signaling pathways^8^. Furthermore, DEGs related to auxin, ethylene and gibberellins during female and male floral differentiation and development were detected in recent study^9^, but the key genes and the regulatory mechanism involved in floral sex differentiation of *J. curcas* were not described. Moreover, the MADS-box gene family – an important floral sex determinant in plants – was also not described^9^.

The MADS-box transcription factors play vital regulatory roles in the floral differentiation process^10^. The core domain structures of these genes are very conservative^11,12^. There are 107 members related to MADS-box genes, which were clustered into class A, B, and C in *Arabidopsis thaliana* and 46 of these members belonged to the type-II subfamily^13^. The genes of the ABC model co-determine the fate of floral organ primordium by complex crosstalk network^14^. Moreover, some genes from class D and E could also join in the network to contribute to male and female floral differentiation. The ABCE model plays role during the floral differentiation of *J. curcas* ^15^. AP1 stimulates flowering and regulates the *B* genes, which allow the differentiation from inflorescence meristem to flower bud^16^. *JcAP1* was shown to trigger early flowering in *Arabidopsis thaliana*, but not *J. curcas* ^17^. The overexpression of *JcFT* in *J. curcas* was shown to induce early flowering^18,19^. However, *JcTFL* represses the flowering of *J. curcas*, and its overexpression in *J. curcas* delays flowering^20^. The crosstalk among *JcAP1*, *JcFT*, and *JcTFL* might co-regulate the flowering time, but the essential genes involved in the differentiation of stamens or pistils of *J. curcas* have not yet been identified.

Moreover, floral differentiation could be regulated by phytohormones, which show complex interaction networks. Gibberellins (GAs) play vital roles during floral differentiation^21^, and their signal transduction could be achieved by degrading the DELLA proteins^22^. Auxins (IAA) have a significant effect on the maturation of female and male flowers^9,23^. Jasmonic acid (JA) regulates the differentiation of floral organs, and the homologs of JA biosynthesis were down-regulated in the gynoecious inflorescences of *J*. *curcas* ^8^. The function of Cytokinins (CTKs) was reversed with GAs such that it could induce female floral differentiation in *Mercurialis annua* ^24^. Brassinolides (BRs) were involved in crosstalk with GAs, and the co-regulated factors were the DELLA proteins^25,26^. Moreover, BRs directly interact with SVP by activating BZR1, which is a new avenue in the floral-regulating network^27^. Plant growth regulators, such as 6-Benzyl aminopurine (6-BA), thidiazuron (TDZ) and paclobutrazol (PAC), could significantly improve female flower formation in *J. curcas* ^28–31^. However, the phytohormone signal transduction pathway during flower sex differentiation in *J. curcas* is still not understood.

Therefore, the present study was carried out to compare five different transcriptomic profiles of floral sex differentiation stages using the Illumina Hiseq 4000 to reveal the underlying regulatory mechanism of male and female floral differentiation. Moreover, the numerous differentially and specifically expressed genes in male and female floral differentiation process were identified. This study were also provides new insights into floral sex differentiation in *J. cur*cas.

## Results

### Morphological observation of flower buds at different developmental stages

**Figure 1:**
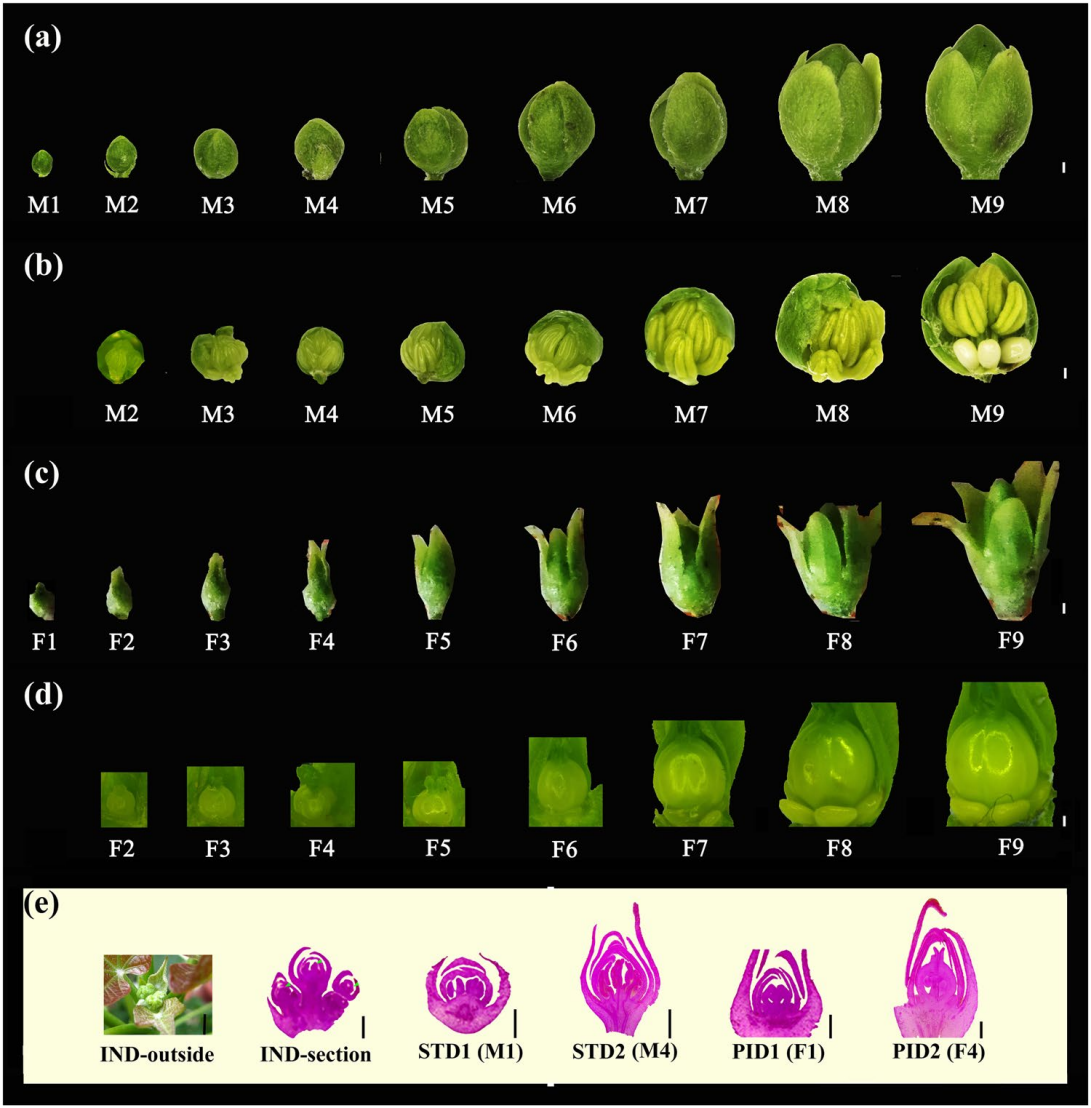
A shows of floral organogenesis and sex differentiation in *J. curcas*. (**a**) the external morphological observation of male floral differentiation process (M1–M9). (**b**) the anatomic observation of male floral differentiation from M2 to M9. (**c**) the external morphological observation of female flower differentiation process (F1–F9). (**d**) the anatomic observation of female floral differentiation from F2 to F9. (**e**) the samples selected for RNA-seq. IND-outside and IND-section were the external morphological observation and longitudinal section of undifferentiated inflorescence (IND, by green arrows), respectively. STD1 (M1) was the longitudinal section of male floral initial stage. STD2 (M4) was the longitudinal section of ten complete stamens formed. PID1 (F1) was the longitudinal section of female floral initial stage. PID2 (F4) was the longitudinal section of complete carpel and ovary formed. The bar was 0.5 cm in IND-outside, and the others were 0.5 mm.

From the morphological observation of male and female floral differentiation process (M1-M9, and F1-F9) as shown in Fig. 1, the male flower buds were tended to develop a spherical shape. Meanwhile, the female flower buds were ellipsoid. Flower buds in the inflorescence stage of 0.5 cm diameter (IND stage) were in the primordial period with no differentiation into stamen and pistil flowers. Five stages of the floral differentiation were selected and defined as stamen primordia beginning to differentiate (STD1) or (M1), ten complete stamens formed (STD2) or (M4), carpel primordia beginning to differentiate (PID1) or (F1), three distinct carpels formed (PID2) or (F4), and undifferentiated inflorescence of 0.5 cm diameter (IND). M1 stage was the initial stage of male floral differentiation. In this stage, the ten stamens were triggered from the male primordial, whereas in the M4 stage, stamens were formed and the complete stamens could be observed. Meanwhile, F1 stage was the initial stage of female floral differentiation and in this stage, the carpel primordial was triggered, while the complete carpel and ovule could be observed in the F4 period. The male and female flowers matured gradually after F4 and M4. Moreover, the accuracy of the connection between the floral exterior structure and interior differentiation stage was more than 93% (Supplementary Table S1).

### Sequence analysis of different cDNA libraries

IND, STD1 (M1), STD2 (M4), PID1 (F1) and PID2 (F4) were selected as different samples of floral sex differentiation stages for transcriptome sequencing (Fig. 1e). Transcriptome sequencing was done using Illumina Hiseq^TM^ 4000 platform and paired-end reads. After trimming adapters and low quality bases, 7.46 G, 6.86 G, 7.84 G, 6.56 G and 7.15 G high quality clean bases were remained for IND, STD1, STD2, PID1 and PID2, respectively (Supplementary Table S2). The error rate of RNA-seq was only 0.02%, all of the Q30 were more than 91%, and the GC content was more than 43% in each sample. In addition, the alignable reads ranged from 35.57 million (STD1, 77.73%) to 40.73 million (IND, 81.94%) based on clean reads (Supplementary Table S3). Among the mapped reads, 34.71–39.88 million were uniquely aligned reads, with IND making up the highest percentage (80.25%).

### Regulatory patterns of male floral differentiation

**Figure 2:**
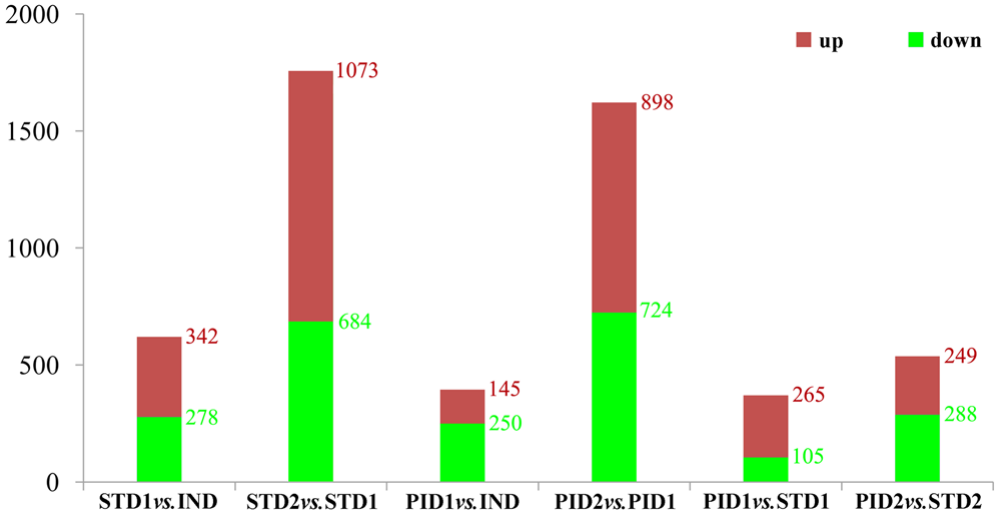
The DEGs statistics during male and female floral differentiation in *J. curcas*. All DEGs were divided into 6 comparisons, STD1 *vs*. IND, STD2 *vs*. STD1, PID1 *vs*. IND, PID2 *vs*. PID1, PID1 *vs*. STD1, and PID2 *vs*. STD2. Red was up-regulated and green was down-regulated.

2,377 DEGs were identified in STD1 *vs*. IND and STD2 *vs*. STD1 (Fig. 2). There were only 620 DEGs identified in STD1 *vs*. IND. However, 1,757 DEGs were detected in STD2 *vs*. STD1. These results suggested that only some few DEGs were needed to initiate male floral differentiation, but numerous DEGs were required for its further development. In addition, a majority of DEGs were up-regulated in male floral differentiation process (Fig. 2).

The expression profiles of each sample related to the 620 DEGs in STD1 *vs*. IND were performed with the normalization method (Supplementary Fig. S1). Most of DEGs up-regulated in STD1 were down-regulated in STD2 and the entire stages of female floral differentiation. This result indicated that these genes were significantly associated with the initiation of male flowers. The down-regulated DEGs in STD1 were still shut down in STD2. However, some of which were activated during female flower differentiation. This result suggested that these genes might be dispensable in the male floral development process, whereas they were required for female floral differentiation.

The expression profiles of each sample related to the 1757 DEGs in STD2 *vs*. STD1were performed with the normalization method (Supplementary Fig. S2). From the up-regulated DEGs in STD2 were down-regulated in STD1 and PID1, its might be suggested that the initiation of floral differentiation was completed and the floral buds had progressed to the stage of stamen organs development. Some co-detected DEGs down-regulated in IND, STD2, PID1 and PID2 were activated in STD1. Its indicated that these genes might be more significantly correlated with the initiation of male floral differentiation, but not associated with the following development events.

**Figure 3:**
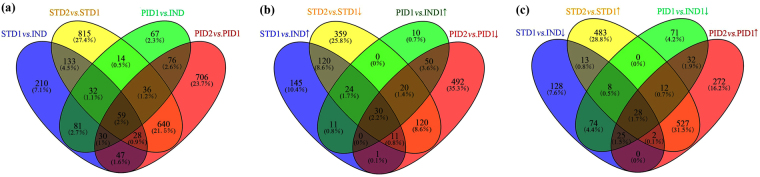
Venn diagrams shared with numbers and proportion of DEGs. (**a**) venn diagram of total DEGs in different samples. (**b**) DEGs were up-regulated in STD1 *vs*. IND and PID1 *vs*. IND, but down-regulated in STD2 *vs*. STD1 and PID2 *vs*. PID1. (**c**) DEGs were down-regulated in STD1 *vs*. IND and PID1 *vs*. IND, but up-regulated in STD2 *vs*. STD1 and PID2 *vs*. PID1.

**Figure 4:**
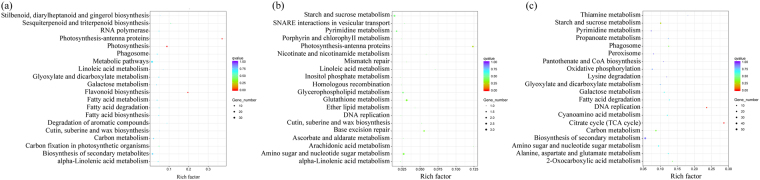
The KEGG enrichment pathways involved in male floral differentiation. The Rich factor indicated the percentages of DEGs belong to the corresponding pathway. The left y-axis represented the enrichment pathways. The sizes of bubble represent the number of DEGs in the corresponding pathway, and the colors of the bubble represent the enrichment Q value of the corresponding pathway. (**a**) KEGG enrichment pathways of the DEGs exclusively detected in STD1 *vs*. IND. (**b**) KEGG enrichment pathways of the DEGs exclusively co-detected in STD1 *vs*. IND and STD2 *vs*. STD1. (**c**) KEGG enrichment pathways of the DEGs exclusively detected in STD2 *vs*. STD1.

There were 210 DEGs exclusively detected in STD1 *vs*. IND (Fig. 3a). Based on the KEGG metabolic pathway analysis (Fig. 4a), six genes (JC04785, JC18282, JC21298, JC06610, JC20688, and JC11710) were significantly enriched in the flavonoids biosynthesis pathway, all of them were up-regulated in STD1 *vs*. IND (Table 1). Moreover, four DEGs, JC11754 (JcSEP1), JC14482 (JcSOC1), JC14484 (JcAGL6) and JC25595 (JcAP1) were annotated with MADS-box transcription factor. JC11754 was up-regulated. Meanwhile, JC14482, JC14484 and JC25595 were down-regulated in STD1 *vs*. IND. In addition, three DEGs: JC06233 (JcIAA19), JC17975 (JcAHK3) and JC19526 (JcARR3), were involved in plant endogenous hormone signal transduction pathway. JC06233 (JcIAA19) was annotated with auxin-activated signaling pathway, whereas JC17975 (JcAHK3) and JC19526 (JcARR3) were annotated with cytokinin-activated signaling pathway (Table 1, Supplementary Fig. S3). Other DEGs were mainly related to the plant photosynthesis, sugar metabolism, and protein synthesis pathways. These exclusive genes may be specifically enriched in STD1 *vs*. IND to trigger the male floral initiation.

**Table 1:** The important DEGs exclusively detected in male floral differentiation process.

**Comparison**	**Gene ID**	**Regulation**	***At*** *.* **name**	**Blastx to TAIR 10 database**
STD1 *vs*. IND	JC04785	up	*CCOAMT*	Flavonoid biosynthetic process
STD1 *vs*. IND	JC18282	up	*PCBER1*	Flavonoid biosynthetic process
STD1 *vs*. IND	JC21298	up	*BAN*	Flavonoid biosynthetic process
STD1 *vs*. IND	JC06610	up	*TT5*	Flavonoid biosynthetic process
STD1 *vs*. IND	JC20688	up	*LDOX*	Flavonoid biosynthetic process
STD1 *vs*. IND	JC11710	up	*TT7*	Flavonoid biosynthetic process
STD1 *vs*. IND	JC11754	up	*SEP1, AGL2*	Flower development
STD1 *vs*. IND	JC14482	down	*AGL20, SOC1*	Controls flowering
STD1 *vs*. IND	JC14484	down	*AGL6*	Carpel maturation
STD1 *vs*. IND	JC25595	down	*AP1, AGL7*	Floral meristem determinacy
STD1 *vs*. IND	JC06233	up	*IAA19*	Auxin-activated signaling pathway
STD1 *vs*. IND	JC17975	down	*AHK3*	Cytokinin-activated signaling pathway
STD1 *vs*. IND	JC19526	down	*ARR3*	Cytokinin-activated signaling pathway
STD2 *vs*. STD1	JC23029	up	*PSBP6*	Photosynthesis
STD2 *vs*. STD1	JC25229	up	*SCPL42*	Serine carboxypeptidase synthesis
STD2 *vs*. STD1	JC11997	up	*AGL104*	Pollen maturation
STD2 *vs*. STD1	JC15741	down	*SEP1, AGL2*	Floral meristem differentiation

All of the genes were annotated with TAIR 10 database. Gene ID is the gene number in RNA-seq database of *Jatropha curcas*. *At*. name is the gene name of homologous gene in *Arabidopsis thaliana*.

133 DEGs were exclusively co-detected in STD1 *vs*. IND and STD2 *vs*. STD1 (Fig. 3a), these genes were specifically associated with the entire male floral differentiation process. However, only two co-detected DEGs (JC23029, JC25229) were up-regulated both in STD1 *vs*. IND and STD2 *vs*. STD1. Based on the BLASTX with TAIR 10 database, JC23029 (*JcPSBP6*) was involved in the plant photosynthesis pathway and JC25229 (*JcSCPL42*) was related to serine carboxypeptidase synthesis pathway (Table 1). Additionally, 118 exclusively co-detected DEGs were up-regulated in STD1 *vs*. IND. However, they were down-regulated in STD2 *vs*. STD1, and mainly related to protein processing, DNA replication, sucrose metabolism pathways (Fig. 4b). There were not any DEGs detected to associate with MADS-box and plant endogenous hormone signal transduction pathway in the exclusively co-detected DEGs of STD1 *vs*. IND and STD2 *vs*. STD1.

Moreover, 815 DEGs exclusively detected in STD2 *vs*. STD1 (Fig. 3a) were mainly involved in plant growth and development pathways (Fig. 4c). 469 DEGs of them were significantly up-regulated to ensure their supporting roles during male floral organ development. Furthermore, two DEGs, JC11997 (*JcAGL104*) and JC15741 (*JcSEP1*), were annotated with MADS-box transcription factor (Table 1). JC11997 was up-regulated and associated with pollen maturation, JC15741 was down-regulated and involved in floral meristem differentiation. There were still no DEGs detected to associate with plant endogenous hormone signal transduction pathway in the exclusively library of STD2 *vs*. STD1.

### Regulatory patterns of female floral differentiation

In this study, 2,017 DEGs were identified in PID1 *vs*. IND and PID2 *vs*. PID1 (Fig. 2). Similar to male flowers, only 395 DEGs were isolated in PID1 *vs*. IND and 1,622 DEGs were detected in PID2 *vs*. PID1. However, the DEGs up-regulated from PID1 *vs*. IND to PID2 *vs*. PID1 (753 DEGs) were more than those in male floral differentiation from STD1 *vs*. IND to STD2 *vs*. STD1 (731 DEGs). In addition, a majority of the DEGs were significantly down-regulated in PID1 *vs*. IND (Fig. 2) and more than 40% DEGs were still down-regulated in PID2 *vs*. PID1.

The expression profiles of each sample related to the 395 DEGs in PID1 *vs*. IND (145 up-regulated and 250 down-regulated) were performed with the normalization method (Supplementary Fig. S4). Actually, 65 genes of the 145 up-regulated DEGs were showed a lower expression in PID1. However, these 65 genes were significantly up-regulated in STD1. Thus, only 80 authentically up-regulated DEGs were detected in PID1. Furthermore, the 80 DEGs were only up-regulated in PID1 *vs*. IND to trigger the female floral initiation. On the contrary with male floral differentiation, the largely down-regulated DEGs in PID1 might be activated in PID2. It was indicated that specific genetic regulatory pathways were operated between female and male floral differentiation in *J. curcas*.

The expression profiles of each sample related to the 1622 DEGs in PID2 *vs*. PID1 were performed with the normalization method (Supplementary Fig. S5). The expression patterns of these DEGs were similar to the male floral development process. The up-regulated DEGs in PID2 were mainly down-regulated in IND, STD1 and PID1. This result was suggested that the initiation of female floral differentiation was complete and floral buds had progressed to the stage of pistil organs development.

**Figure 5:**
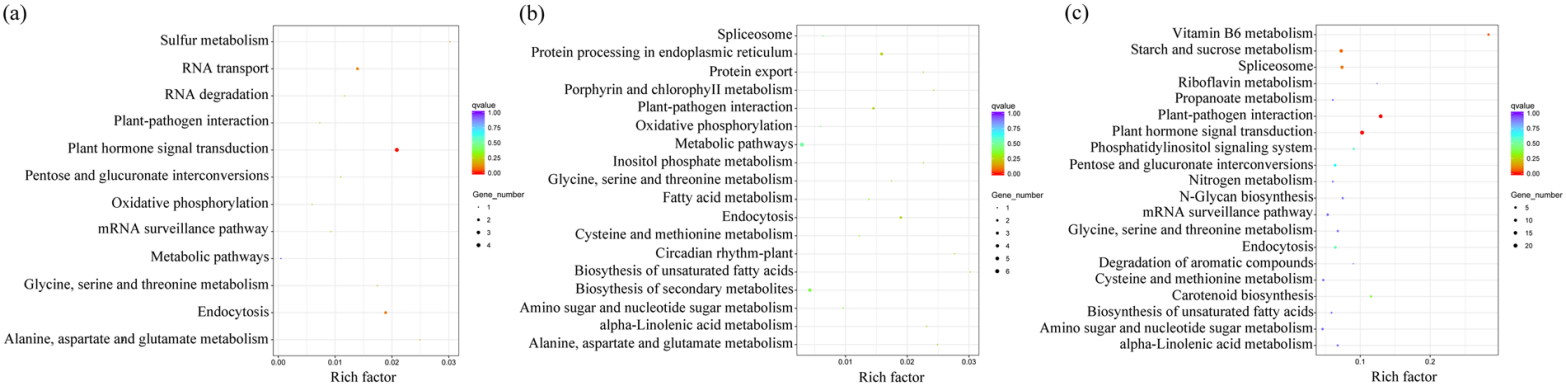
The KEGG enrichment pathways associated with female floral differentiation. The Rich factor indicated the percentages of DEGs belong to the corresponding pathway. The left y-axis represented the enrichment pathways. The sizes of bubble represent the number of DEGs in the corresponding pathway, and the colors of the bubble represent the enrichment Q value of the corresponding pathway. (**a**) KEGG enrichment pathways of the DEGs exclusively detected in PID1 *vs*. IND. (**b**) KEGG enrichment pathways of the DEGs exclusively co-detected in PID1 *vs. vs*. IND and PID2 *vs*. PID1. (**c**) KEGG enrichment pathways of the DEGs exclusively detected in PID2 *vs*. PID1.

67 DEGs were exclusively detected in PID1 *vs*. IND (Fig. 3a). Based on the KEGG metabolic pathway analysis (Fig. 5a), these 67 DEGs were significantly enriched in the phytohormone signal transduction pathway. Four genes, JC09441 (*JcBRI1*), JC01328 (*JcCOI1*), JC25828 (*JcGH3*), JC23402 (*JcAHP1*) were involved in BR signaling, JA signaling, IAA signaling, and CTK signaling pathways, respectively (Table 2, Supplementary Fig. S3), all of them were down-regulated in PID1 *vs*. IND. Other DEGs were mainly related to the RNA transport pathways. These genes were specifically enriched in PID1 *vs*. IND to induce the female floral initiation. However, no genes were detected to associate with MADS-box transcription factor in the 67 DEGs exclusively detected in PID1 *vs*. IND.

**Table 2:** The vital DEGs exclusively detected in female floral differentiation process.

**Comparison**	**Gene ID**	**Regulation**	***At*** *.* **name**	**Blastx to TAIR 10 database**
PID1 *vs*. IND	JC01328	down	*COI1*	Jasmonic acid signaling pathway
PID1 *vs*. IND	JC09441	down	*BRI1*	Brassinosteroid signaling pathway
PID1 *vs*. IND	JC23402	down	*AHP1*	Cytokinin signaling pathway
PID1 *vs*. IND	JC25828	down	*GH3*	Response to auxin
PID2 *vs*. PID1	JC01392	down	*PP2CA*	Negative regulation of ABA signaling pathway
PID2 *vs*. PID1	JC02934	down	*HAI2*	Negative regulation of ABA signaling pathway
PID2 *vs*. PID1	JC04255	down	*ABF2*	Negative regulation of ABA signaling pathway
PID2 *vs*. PID1	JC04805	down	*CRE1*	Cytokinin-activated signaling pathway
PID2 *vs*. PID1	JC07511	down	*JAZ2*	Negative regulation of JA signaling pathway
PID2 *vs*. PID1	JC11537	down	*ARR5*	Cytokinin-activated signaling pathway
PID2 *vs*. PID1	JC14204	down	*MYC4*	Jasmonic acid mediated signaling pathway
PID2 *vs*. PID1	JC21755	down	*ARR3*	Cytokinin-activated signaling pathway
PID2 *vs*. PID1	JC23114	down	*JAZ10*	Negative regulation of JA signaling pathway
PID2 *vs*. PID1	JC24672	down	*JAZ1*	Negative regulation of JA signaling pathway
PID2 *vs*. PID1	JC25989	down	*HAI2*	Negative regulation of ABA signaling pathway
PID2 *vs*. PID1	JC26194	down	*AHG1*	Negative regulation of ABA signaling pathway
PID2 *vs*. PID1	JC02272	up	*IAA14*	Auxin-activated signaling pathway
PID2 *vs*. PID1	JC07991	up	*STK, AGL11*	Carpel development
PID2 *vs*. PID1	JC12057	up	*LAX3*	Auxin polar transport
PID2 *vs*. PID1	JC13432	up	*RGA1*	Gibberellic acid mediated signaling pathway
PID2 *vs*. PID1	JC19628	up	*SAUR-like*	Response to auxin
PID2 *vs*. PID1	JC23499	up	*IAA4*	Auxin-activated signaling pathway

In addition, 76 DEGs were exclusively detected in PID1 *vs*. IND and PID2 *vs*. PID1 (Fig. 3a). Interestingly, 45 DEGs were up-regulated in PID1 *vs*. IND and down-regulated in PID2 *vs*. PID1, while the rest 31 genes were down-regulated in PID1 *vs*. IND and up-regulated in PID2 *vs*. PID1. No DEG was continuously up or down regulated both in PID1 *vs*. IND and PID2 *vs*. PID1. These results were indicated that the female floral sex initiation and development needed a more complex regulatory mechanism compared with male floral differentiation in *J. curcas*. Similarly with the exclusively co-detected DEGs in the male floral differentiation process, the 76 DEGs were also annotated to protein synthesis and plant growth pathways (Fig. 5b), and no DEGs was involved in MADS-box and plant endogenous hormone signal transduction pathway.

Moreover, 706 DEGs were exclusively detected in PID2 *vs*. PID1. 18 genes of them were significantly annotated to the plant endogenous hormone signal transduction pathway (Fig. 5c), including IAA signal (JC02272, JC12057, JC16280, JC19628, JC23499), ABA signal (JC01392, JC02934, JC04255, JC25989, JC26194), CTK signal (JC04805, JC11537, JC21755), GA signal (JC13432), and JA signal (JC07511, JC14204, JC23114, JC24672) transduction pathways (Table 2). However, the DEGs involved in the CTK signal transduction pathway were down-regulated, and the IAA, ABA, GA, and JA signal transduction pathway were activated (Table 2, Supplementary Fig. S3). Only one MADS-box transcription factor was detected in these exclusively DEGs, JC07991 (*JcSTK*), which was related to carpel development. Other DEGs were mainly related to sucrose metabolism, nucleotide sugar metabolism, and protein synthesis pathways. Therefore, the transduction of phytohormones also played vital roles in female floral development process.

Also 370 DEGs were detected in PID1 *vs*. STD1 (Fig. 2). Among the 370 DEGs, 265 DEGs were up-regulated, whereas 105 DEGs were down-regulated. Based on the KEGG enrichment analysis, the up-regulated DEGs were mainly related to endogenous hormone signal transduction pathways. However, the down-regulated DEGs were significantly involved in the flavonoid biosynthesis pathway (Supplementary Fig. S6). Furthermore, CTK and IAA signal transduction pathway were activated, but the ABA and JA signal transduction pathway were negative regulation in PID1 *vs*. STD1 (Table 3). In addition, 537 DEGs were detected in PID2 *vs*. STD2. 249 DEGs from the 537 DEGs were up-regulated, meanwhile 288 DEGs were down-regulated (Fig. 2). The up-regulated DEGs were still significantly related to plant endogenous hormone signal transduction pathways (Supplementary Fig. S7) such as five of them were involved in IAA signal (JC18784), GA signal (JC13432), ETH signal (JC07165), BR signal (JC22124), and SA signal (JC06956) pathways (Table 3, Supplementary Fig. S3). The down-regulated DEGs were still related to the carbohydrate metabolism and flavonoids biosynthesis pathway. Thus, the phytohormones significantly regulated the female floral initiation and development process, whereas the flavonoids biosynthesis pathway was significantly involved in male floral initiation and development process.

**Table 3:** The significant DEGs detected in PID1 *vs*. STD1 and PID2 *vs*. STD2.

**Comparison**	**Gene ID**	**Regulation**	***At*** *.* **name**	**Blastx to TAIR 10 database**
PID1 *vs*. STD1	JC04459	down	*TT3*	Flavonoid biosynthetic process
PID1 *vs*. STD1	JC06413	down	*TT3*	Flavonoid biosynthetic process
PID1 *vs*. STD1	JC06540	down	*TT4*	Flavonoid biosynthetic process
PID1 *vs*. STD1	JC06610	down	*TT5*	Flavonoid biosynthetic process
PID1 *vs*. STD1	JC11710	down	*TT7*	Flavonoid biosynthetic process
PID1 *vs*. STD1	JC21298	down	*BAN*	Flavonoid biosynthetic process
PID1 *vs*. STD1	JC26889	down	*TT4*	Flavonoid biosynthetic process
PID1 *vs*. STD1	JC01392	up	*PP3CA*	Negative regulation of ABA signaling pathway
PID1 *vs*. STD1	JC04255	up	*ABF3*	Negative regulation of ABA signaling pathway
PID1 *vs*. STD1	JC07511	up	*JAZ3*	Negative regulation of JA signaling pathway
PID1 *vs*. STD1	JC11537	up	*ARR6*	Cytokinin-activated signaling pathway
PID1 *vs*. STD1	JC16280	up	*GH3.2*	Response to auxin
PID1 *vs*. STD1	JC23114	up	*JAZ11*	Negative regulation of JA signaling pathway
PID1 *vs*. STD1	JC24672	up	*JAZ2*	Negative regulation of JA signaling pathway
PID1 *vs*. STD1	JC25989	up	*HAI3*	Negative regulation of ABA signaling pathway
PID2 *vs*. STD2	JC06956	up	*PR1*	Defense response
PID2 *vs*. STD2	JC07165	up	*EBF1*	Ethylene-activated signaling pathway
PID2 *vs*. STD2	JC13432	up	*RGA1*	Gibberellic acid mediated signaling pathway
PID2 *vs*. STD2	JC18784	up	*LAX2*	Auxin-activated signaling pathway
PID2 *vs*. STD2	JC22124	up	*CYCD3*	Brassinosteroid and cytokinin response

### DEGs analysis involved in floral differentiation and development

According to the DEG analysis of our five samples, 30 genes were up-regulated both in STD1 *vs*. IND and PID1 *vs*. IND, whereas down-regulated both in STD2 *vs*. STD1 and PID2 *vs*. PID1 (Fig. 3b). One of them was annotated to MADS-box transcription factor, JC12152 (*JcPI*), which was related to the petal development (Table 4). Moreover, two of them, JC13576 (*JcTGA9*) and JC25510 (*JcTGA10*), were involved in SA signal transduction pathways (Supplementary Fig. S3). These DEGs were associated with the initiation of floral primordia, but they have no significant roles in floral sex determination and subsequent development process. In addition, 28 genes were down-regulated both in STD1 *vs*. IND and PID1 *vs*. IND, whereas up-regulated both in STD2 *vs*. STD1 and PID2 *vs*. PID1 (Fig. 3c). These genes were mainly related to energy supplement in subsequent floral development to ensure the normal differentiation of floral organs. Only JC14209 annotated with seed development process (*JcCYP78A9*) was significantly down-regulated in the entire stages of female and male floral differentiation. There were not any DEGs up-regulated in all comparisons. These results indicated that some genes were common in male and female floral initiation or development process to play similar roles in *J. curcas*.

**Table 4:** The DEGs associated with MADS-box transcription factor.

**Gene ID**	***At*** *.* **locus**	***At*** *.* **name**	**Blastx to TAIR 10 database**
JC00165	AT3G58780.1	*SHP1, AGL1*	Carpel development
JC04507	AT3G54340.1	*AP3*	Petal and stamen development
JC07594	AT1G18750.1	*AGL65*	Pollen tube growth
JC07991	AT4G09960.3	*STK, AGL11*	Carpel development
JC11754	AT5G15800.1	*SEP1, AGL2*	Flower development
JC11997	AT1G22130.1	*AGL104*	Pollen maturation
JC12153	AT5G20240.1	*PI*	Petal identity
JC13660	AT3G54340.1	*AP3*	Petal and stamen development
JC14482	AT2G45660.1	*AGL20, SOC1*	Positive regulation of flower development
JC14484	AT2G45650.1	*AGL6*	Carpel maturation
JC15741	AT5G15800.1	*SEP1, AGL2*	Floral meristem differentiation
JC15742	AT1G69120.1	*AP1, AGL7*	Floral meristem determinacy
JC17987	AT1G24260.1	*SEP3, AGL9*	Floral meristem differentiation
JC18099	AT3G54340.1	*AP3*	Petal and stamen development
JC18694	AT2G22540.1	*SVP, AGL22*	Floral meristem determinacy
JC18739	AT2G22540.1	*SVP, AGL22*	Floral meristem determinacy
JC21634	AT4G18960.1	*AG*	Carpel development
JC24097	AT1G22130.1	*AGL104*	Pollen development
JC25593	AT5G15800.1	*SEP1, AGL2*	Floral meristem differentiation
JC25595	AT1G69120.1	*AP1, AGL7*	Floral meristem determinacy
JC26434	AT1G26310.1	*CAL1*	Floral meristem determinacy

*At*. locus is the locus of homologous gene in *Arabidopsis thaliana*.

### DEGs analysis related to the MADS-box transcription factors

In a previous study, the MADS-box genes play vital regulatory roles in the floral differentiation process. Among the DEGs in our comparisons, 21 DEGs related to MADS-box transcription factors were isolated in male and female floral differentiation process (Table 4). Furthermore, these genes were only significantly up-regulated at one stage (IND, STD1, STD2, PID1 or PID2) (Supplementary Fig. S8). Seven of these genes were up-regulated in IND, 8 in STD1, 2 in STD2, and 4 in PID2; however, none of them were significantly up-regulated in PID1.

**Figure 6:**
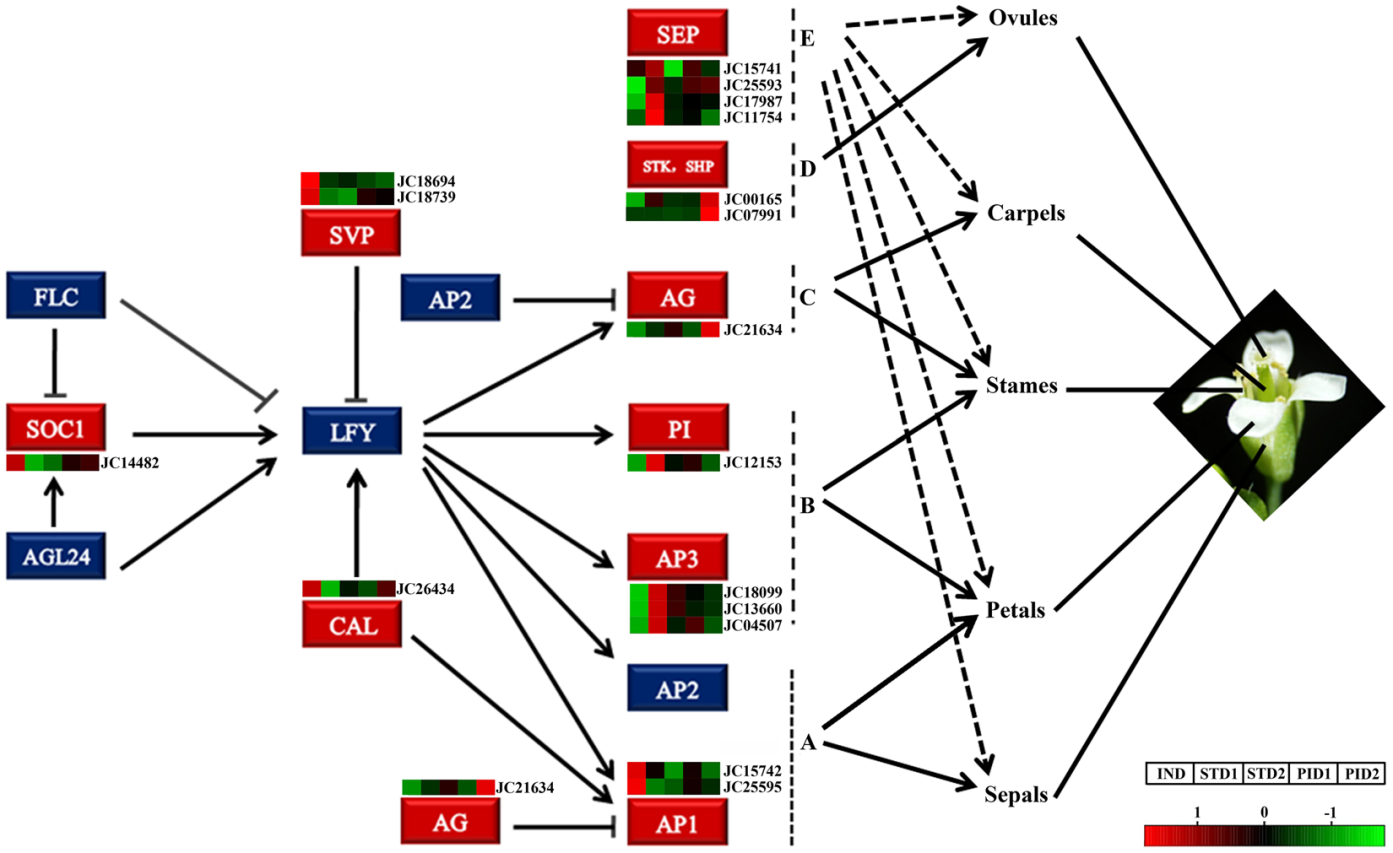
The regulatory cascade of floral differentiation related to the MADS-box transcription factors in *Arabidopsis*. Red box indicates the homologous gene differentially expressed in *J. curcas*. Blue box indicates the genes without differentially expressed in present study. Heat map is the DEGs involved in MADS-box transcription factor obtained by RNA-seq. Z-score was calculated by scale package of R software using FPKM of different samples. Red was up-regulated and green was down-regulated.

Based on the BLASTX with TAIR 10 database (Table 4, Fig. 6), JC07594 (*JcAGL65*) was mainly involved in pollen tube growth. JC18694 and JC18739 (*JcAGL22/SVP*), JC25595 and JC15742 (*JcAGL7/AP1*), JC14482 (*JcAGL20/SOC1*), and JC26434 (*JcAGL10/CAL1*) were mainly related to the transition from vegetative to reproductive period in IND. Most of DEGs associated with MADS-box transcription factors were detected in STD1, but were only separated in four clusters, including *JcAGL2/SEP1* (JC11754, JC15741, and JC25593), *JcAGL9/SEP3* (JC17987), *JcATAP3/AP3* (JC04507, JC13660, and JC18099), and *JcPI* (JC12153). They played the key roles in male floral initiation. Two DEGs annotated to *JcAGL104* (JC11997, JC24097) were not only up-regulated in STD2, but also involved in stamen development and maturation. Four DEGs: JC00165, JC07991, JC14484 and JC21634, were significantly up-regulated in PID2. They were annotated to *JcAGL1/SHP1*, *JcAGL11/STK*, *JcAGL6* and *JcAG* respectively, all of them were involved in carpel maturation.

### qRT-PCR validation

**Figure 7:**
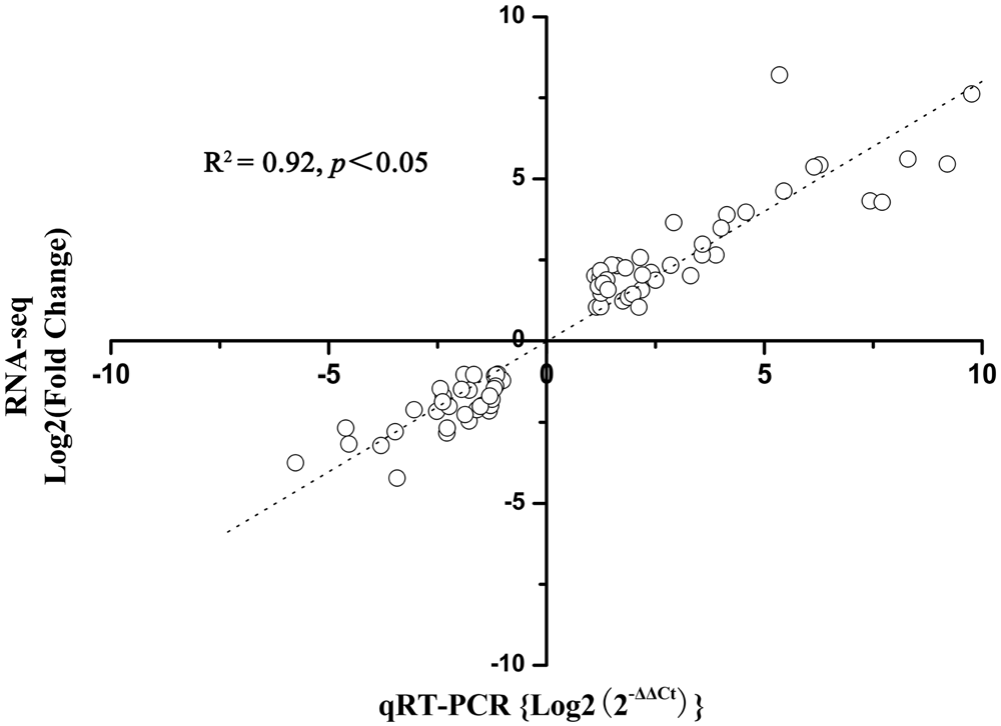
The correlation between qRT-PCR and RNA-seq data. Correlation between qRT-PCR and RNA-seq data of 74 selected genes: 39 up-regulated genes and 35 down-regulated genes in 6 pairs of amplified RNA samples. Spearman Rank Correlation coefficient = 0.92 (*P* < 0.05).

To validate the RNA-seq data, 74 DEGs identified by RNA-seq were tested by qRT-PCR (Supplementary Table S4). These genes were selected because of their important function in floral sex differentiation process, including 39 up-regulated genes and 35 down-regulated genes. All of them were consistent with the same trend of up- or down- regulation between the two different expression analysis platforms (Fig. 7). The correlation of the two expression measurements was 0.92 between these 74 genes (*R* ^2 = ^0.92). In fact, the results of RNA-seq and qRT-PCR were consistent.

## Discussion

The present study showed that some key specific genes were activated in STD1 or PID1 to trigger male or female floral initiation, but they were shut down in the subsequent development process. After floral sex determination, the numerous DEGs were activated to complete the development of stamens or ovules. Similar regulated events were observed in gymnosperms and lower angiosperms^32–34^, which indicated that this regulated pattern is conserved in plants. However, the down-regulated DEGs in STD1 were also down-regulated in STD2, but the down-regulated DEGs in PID1 could be activated in PID2. Thus, female floral differentiation was a more complex set compared with male floral differentiation.

In previous studies, the numerous down-regulated DEGs were attributable to the gynoecious mutation against with monoecious wild-type in Cucumber^35^. The similar result in our study was that the DEGs were mainly up-regulated in male floral differentiation process, but down-regulated in female floral initiation. However, the DEGs up-regulated from PID1 *vs*. IND to PID2 *vs*. PID1 were more than those in the male flower differentiation from STD1 *vs*. IND to STD2 *vs*. STD1 (Fig. 2). Based on the scanning electron microscope observation, the floral differentiation process was divided into 12 stages in *J. curcas* ^5^. The floral primordium is originally differentiated to male flowers from first to sixth stage, but it might be reversed to female floral differentiation by some endogenous induction effects in the seventh stage. As a result of which, the male floral primordium underwent abortion gradually in the subsequent development process. Recently, the certain genes including *CUC2, TAA1, CKX1*, and *PIN1* promote the formation of female flowers in *J. curcas* while the genes *SUP* and *CRY2* are involved in female flower transition and male primordia abortion^36^. In addition, the differentiation of arrested pistil primordia could be activated in male flowers by repressing the expression of the *TS2* which is required for carpel abortion in *J. curcas* ^37^. Thus, we suggested that some genes could continuously inhibit the initiation of female floral primordium in the original period of floral differentiation in *J. curcas*. If these genes were down-regulated to release the inhibition in the floral sex determination point, the female floral differentiation would be triggered, thereafter numerous genes would be activated in the subsequent development process, and visa versa. However, not all primordia revert to female flowers. Therefore, both male and female flowers could be present in the same inflorescence. Furthermore, the released location of the inhibition might be the female floral site observed in the present study. It was also revealed the underlying reason why the poor female flower was showed in the inflorescence of *J. curcas*. In further study, a strategic approach is to find the key genes related to the inhibition and releasing, and then express them with GUS protein to confirm their expressed location and function in the inflorescence. From this study, the key inhibited genes could also be knocked down to improve the female flower and increase the seed yield of *J. curcas*.

Additionally, male floral differentiation was significantly associated with flavonoid biosynthesis process in the present study. The inhibition of flavonoid biosynthesis causes male sterility in Petunia^38^, but the same results could not be obtained in *Arabidopsis thaliana* ^39^. This suggested that flavonoids may play various roles in different species. Thus, the *Jatropha* flavonoid pathways should be further explored to understand the regulatory events involved in male floral differentiation. Moreover, phytohormones were required to trigger and maintain the female floral initiation and development in present study. Several studies showed that female flowers could be induced by different plant growth regulators to improve the fruit yield in *J. curcas* ^29–31,40^, the further research should focus on the regulation of endogenous plant hormone for floral sex differentiation in *J.curcas*.

Phytohormones and their crosstalk were played vital roles in the floral sex differentiation process. Some genes related to IAA, ETH and GAs have been previously isolated during female and male floral development in *J. curcas* ^9^. This study corroborated these results with further detected many other DEGs related to floral sex initiation and development in all of the phytohormone pathways (Supplementary Fig. S3).

IAA plays a significant role both in female and male floral development^9,23,41^. In this study, DEGs involved in the IAA signal transduction pathway were significantly up-regulated in male and female floral development process. A previous study showed that exogenous CTKs could significantly induce the formation of female flowers in *J. curcas* ^28,30^. In this research, we detected six DEGs associated with CTK signaling transduction pathway. JC11537 were significantly up-regulated in PID1 *vs*. STD1. However, JC23403 were down-regulated in PID1 *vs*. IND. Meanwhile, JC04805, JC11537, and JC21755 were down-regulated in PID2 *vs*. PID1, whereas JC17975 and JC19526 were down-regulated in STD1 *vs*. IND (Tables 1, 2). It was indicated that CTK was associated with the initiation of female floral primordium, but not promoted its development. JA play essential roles in regulating the differentiation of floral organs and the homologs of JA biosynthesis were down-regulated in gynoecious inflorescences of *J*. *curcas* ^8^. However, our data showed that the DEGs related to JA signal transduction were significantly up-regulated in PID1 *vs*. STD1 (Table 3), and down-regulated in PID2 *vs*. PID1 (Table 2). Because these genes were mainly annotated with Jasmonate-ZIM-domain (JAZ) which was the negatively regulated factor in JA signal transduction^42,43^. Thus, a higher concentration of JA could inhibit female floral initiation, but promote its development. BRs could crosstalk with GAs to play in the floral-regulating network, and the co-regulated factor was the DELLA protein^21,25,26^.

In the present study, the DEGs involved in the BR signaling transduction pathway were significantly down-regulated in PID1 *vs*. IND, and up-regulated in PID2 *vs*. STD2 (Table 2), indicating that BRs could also play the role to promote the female floral development in *J. curcas*. GA signaling is an important pathway associated with flowering by activating the genes from the MADS-box gene family, such as *SOC1*, *LFY*, and *FT* ^44,45^. Previous studies have shown that the female flowers were induced by spraying exogenous GAs on the inflorescence, but the highest concentration of GAs resulted in the withering of the inflorescence^40^. In the present study, JC13432 involved in the GA mediated signaling pathway was up-regulated in PID2 *vs*. STD2 and PID2 *vs*. PID1. It indicated that GA was also benefit to promote the female floral development in *J. curcas*. ABA signaling transduction could be repressed by exogenous 6-BA treatment which could significantly increase the female flowers and seed yield in *J. curcas* ^37^. In this study, the ABA signal associated with the female floral differentiation played in the negative regulation pathway (Tables 2, 3). The DEGs involved in negative regulation of the ABA signaling pathway were up-regulated in PID1 *vs*. STD1, but down-regulated in PID2 *vs*. PID1. It indicated that ABA signal transduction pathway was significantly involved in female floral development process.

Overall, the regulation of endogenous plant hormone acted as the vital roles for female floral differentiation in *J.curcas*, but not significantly involved in male floral differentiation. We suggested that CTK signaling triggered the initiation of female floral primordium, thereafter other phytohormones co-promoted the female floral development, including JA, BR, GA and ABA. In addition, IAA played the supporting roles in the entire floral differentiation process. This was an evidence to explain the reason that some endogenous induction effects could reverse the floral primordium to female floral differentiation in previous studies. It also revealed the underlying reason that exogenous cytokinin could induce the female floral differentiation and significantly increase the seed yield in *J. curcas*. A further study could focus on measuring the phytohormone concentrations during the male and female floral differentiation using high performance liquid chromatography – electrospray ionization tandem mass spectrometry (HPLC-MS) to verify the induction of endogenous plant hormone for floral sex differentiation and find the better exogenous plant growth regulator to increase the seed yield in *J. curcas*.

In previous studies, the floral organ identity genes known as the ABCE model genes and the transcription factors such as *JcAP-like*, *JcPI*, *JcAG*, and *JcSEP -like* were screened in *J. curcas* ^15^. These genes have been identified to play specific roles in different plants^46–50^, but their function has not been completely elucidated in *J. curcas*. In this study, we have isolated 21 DEGs related to MADS-box transcription factors during female and male floral differentiation. *JcSEP-like*, *JcPI*, and *JcAP3* were significantly up-regulated in STD1 and were associated with the development of petals and stamens^51^. *JcAG* was significantly up-regulated in PID2 and was primarily related to the carpel differentiation. Moreover, *STK-like* were also isolated, its belonging to the class D of the MADS-box transcription factors, which were implicated in carpel maturation^52^, so that the floral differentiation model of *J. curcas* could be extended from the ABCE model to the ABCDE model (Fig. 6). These genes are crucial for studying the molecular mechanism of male and female floral differentiation. Future studies should focus on confirming their molecular function in *J. curcas*, and then overexpress them with transgenic technology to improve the female floral ratio and obtain the high yielding materials.

## Conclusion

The present study revealed the regulated mechanism of floral sex differentiation in *Jatropha curcas* L.using RNA-seq. Our results showed that the initiation of male and female floral primordium just needed some few key genes, but there were numerous DEGs to be activated to complete the development of stamens and ovules. The underlying reason of poor female flower is that the differentiation of female flowers was inhibited to promote the formation of male flowers in floral primordium period. Furthermore, the male floral differentiation was significantly associated with flavonoid biosynthesis process, but female floral differentiation was significantly involved in the phytohormone signal transduction pathway. CTK signaling triggered the initiation of female floral primordium, thereafter other phytohormones co-promoted the female floral development, including JA signaling, BR signaling, GA signaling and ABA signaling. In addition, the floral organ identity genes played important roles in floral sex differentiation and displayed a general conservation of the ABCDE model in *J. curcas*. Moreover, many differentially and specifically expressed genes were screened in male and female floral differentiation process, which were crucial for studying the molecular mechanism of male and female floral differentiation to breed high-yielding *Jatropha* germplasms in the further study.

This data will create a reference transcriptome for the genomics database of *J. curcas* for future studies. Our study will contribute to the understanding of the underlying regulatory mechanism of floral sex differentiation, and the data set will serve as a foundation to study the genes function, which help in engineering high-yielding varieties in *J. curcas*.

## Materials and Methods

### Morphological observation of flower buds at different developmental stages

Male and female flowers occur at a specific location on the *J. curcas* plants. The top of the main inflorescence rachis and the central location, usually the female sites, triggered the formation of female flowers (Supplementary Fig. S9), while the male flowers were formed at the end of the floral branch^5^. In the present study, flower buds were divided into nine stages, based on size, from primordium to mature male or female flowers (M1-M9 and F1-F9). Additionally, 30 floral buds were used to confirm the connection between floral exterior structure and interior differentiation stage respectively. And then, five stages of the floral differentiation were selected and defined as: stamen primordia beginning to differentiate (STD1), ten complete stamens formed (STD2), carpel primordia beginning to differentiate (PID1), three distinct carpels formed (PID2), and undifferentiated inflorescence of 0.5 cm diameter (IND). These stages were identified by stereoscope and paraffin section and sequenced by RNA-seq, respectively.

### Floral samples collection

The *J. curcas* clone of Nujiang in Yunnan province (25.85°N, 98.85°E) was selected as the experimental material, which was planted in a forestry trial base of South China Agricultural University (23.24°N, 113.64°E). Five mixed samples with ten plants, IND, STD1, STD2, PID1 and PID2, were collected respectively, and flash frozen in liquid nitrogen and stored at −80 °C until further use for RNA extraction.

### Total RNA extraction and transcriptome sequencing analysis

Total RNA from all samples was extracted separately using the TIANDZ Plant RNA Kit (TIANDZ column type RNAout 2.0, Beijing). The quantity and purity of total RNA were examined using agarose gel electrophoresis and Nanodrop 2100 (Agilent, USA). The five cDNA libraries in our study, including IND, STD1, STD2, PID1, and PID2 were constructed and sequenced using the Illumina HiSeq™ 4000 (Illumina, USA) platform at Novegene Bioinformatics Technology Co. Ltd (Beijing, China). Sequence adaptors, N’s more than 10% and low-quality reads (Qphred ≤ 20 for >50% read) were removed^53^. When the quality control (QC) finished, the clean reads was mapped to the genome sequence in the NCBI used Tophat^54^ (http://www.ncbi.nlm.nih.gov/genome/?term=Jatropha%20curcas), then the expression abundance were calculated (used HTSeq) and normalized to the expected number of fragments per kilobase of transcript sequence per million fragments mapped (FPKM)^55^.

### Identification and annotation of DEGs

All DEGs were divided into 6 comparisons (STD1 *vs*. IND, STD2 *vs*. STD1, PID1 *vs*. IND, PID2 *vs*. PID1, PID1 *vs*. STD1, PID2 *vs*. STD2). All of the up- or down- regulated genes in the following description were regulated in the first comparison component. Differential expression analysis of each comparison was performed using the DEGSeq R package (1.20.0). Fold change (FC) is the gene expression difference between different samples. We used the threshold |log2 (FC)|>1 and q < 0.005 as the criteria for identifying the DEGs. Functional enrichment and classification of DEGs were performed according to the Kyoto Encyclopedia of Genes and Genomes database (KEGG, http://www.kegg.jp/). KOBAS 2.0 software was used to estimate the statistical enrichment of DEGs in KEGG pathways^56^. Corrected *p*-value of 0.05 was set as the threshold for the significant enrichment of KEGG pathways. Transcription factor prediction analysis was performed by iTAK software (iTAK 1.2)^57,58^. The Blastx alignment was carried out between the DEGs and the TAIR 10 database (https://www.arabidopsis.org/). The heat maps in this study were drawn by R software (R-2.15.3-win), and the normalization method was performed using scale package^59^.

### Quantitative real-time PCR (qRT-PCR) validation

To validate the transcriptomic results, we isolated total RNA from the same plant materials, and a total of 74 DEGs were selected for qRT-PCR analysis. These genes were selected because of their important function in floral sex differentiation process according to DEGs analysis in the present study. The cDNA synthesis for the five stages (IND, STD1, STD2, PID1 and PID2) was performed using PrimeScript^®^ II first Strand cDNA Synthesis Kit (TaKaRa, Japan). The specific primers were designed by Primer Premier 5.0 (Supplementary Table S5). qRT–PCR was performed on the Roche LightCyler480 system (Roche, Germany) with SYBR Premix Ex Taq^TM^ II (TaKaRa, Japan)^60^. The cycling reaction was 94 °C for 2 min, followed by 40 cycles of 94 °C for 10 s, 55 °C for 10 s and 72 °C for 20 s. Three replicates were included for each gene, and *β-*actin (*Jcactin*) and *JcGAPDH* were used as internal controls^61^. The 2^−ΔΔCt^ method was used to calculate the relative expression level of DEGs^62^.

## Electronic supplementary material

Supplementary Information
