# Supplementary material for: Transcriptome profile analysis reveals the regulation mechanism of floral sex differentiation in Jatropha curcas L
Source: Sci Rep. 2017 Nov 27;7:16421. doi: 10.1038/s41598-017-16545-5 (PMC5703882; doi:10.1038/s41598-017-16545-5)
Supplement: Supplementary file 1 — Supplementary Information [file 41598_2017_16545_MOESM1_ESM.pdf]

Supplementary Information

Transcriptome profile analysis reveals the regulation mechanism of floral sex differentiation in *Jatropha curcas* L.

Wenkai Hui[1], Yuantong Yang[2], Guojiang Wu[3], Changcao Peng[2], Xiaoyang Chen[1, 2 *], Mohamed Zaky Zayed[2,4]

1 National Engineering Laboratory for Forest Tree Breeding, College of Biological Science and Technology, Beijing Forestry University, Beijing, 100083, P R. China

2 State Key Laboratory for Conservation and Utilization of Subtropical Agro-bioresources, Guangdong Key Laboratory for Innovative Development and Utilization of Forest Plant Germplasm, College of Forestry and Landscape Architecture, South China Agricultural University, Guangzhou, 510642, P R. China

3 Key Laboratory of Plant Resources Conservation and Sustainable Utilization, South China Botanical Garden, Chinese Academy of Sciences, Guangzhou, 510650, P R. China

4 Forestry and Wood Technology Department, Faculty of Agriculture (EL-Shatby), Alexandria University, Alexandria, Egypt.

* Correspondence: 815849477@qq.com (X.Y. Chen)

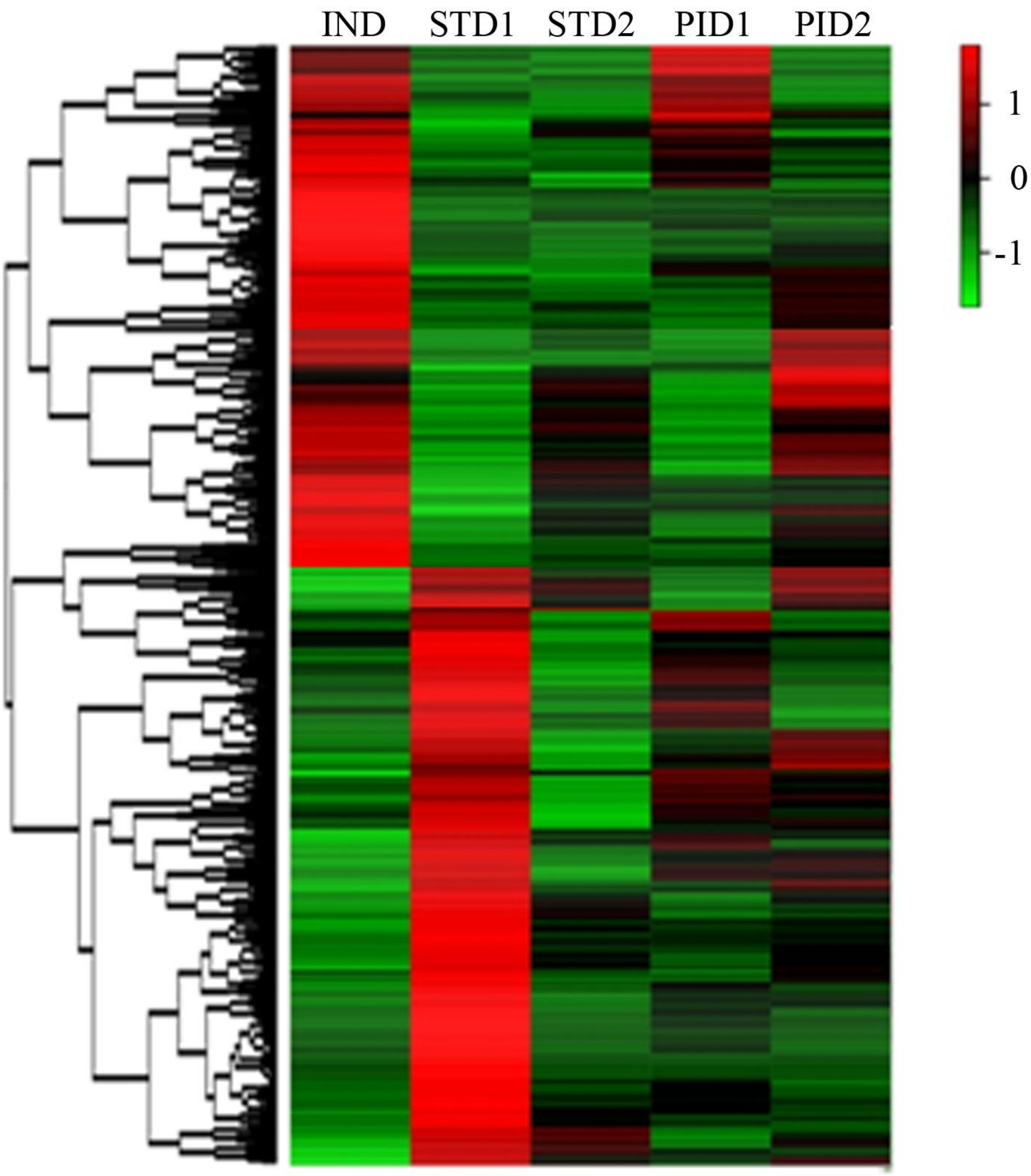

Supplementary Fig. S1 The expression profiles of male and female floral differentiation process related to DEGs in STD1*vs*.IND. Each column contained 620 DEGs detected in STD1*vs*.IND. Each row was the expression in male and female floral differentiation process with the normalization method. Red is up-regulated and green is down-regulated.

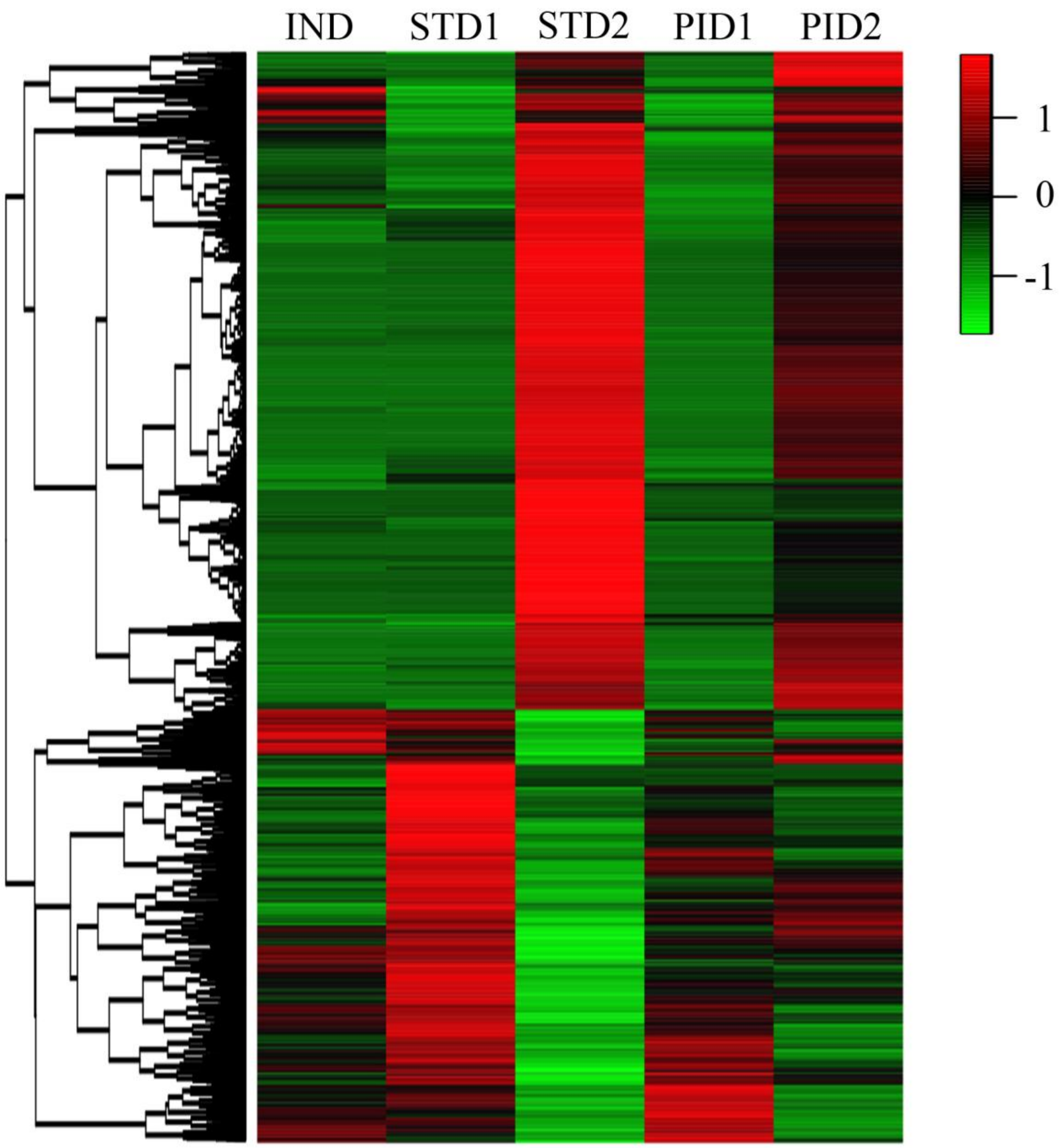

Supplementary Fig. S2 The expression profiles of male and female floral differentiation process related to DEGs in STD2*vs*.STD1. Each column contained 1,757 DEGs detected in STD2*vs*.STD1. Each row was the expression profiles in male and female floral differentiation process with the normalization method. Red is up-regulated and green is down-regulated.

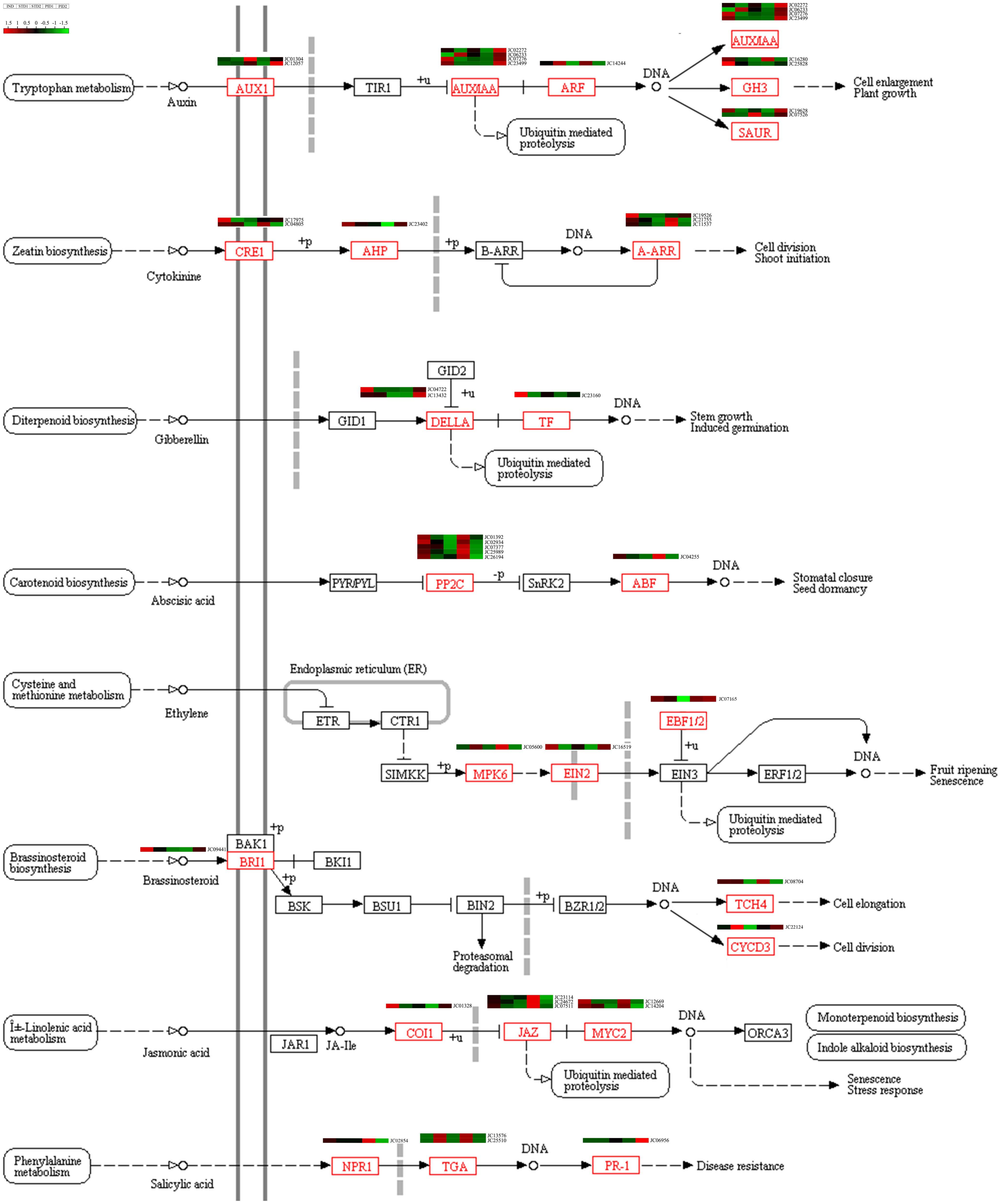

Supplementary Fig. S3 The profiles of DEG involved in phytohormone signaling transduction pathway. The expression of DEGs in male and female floral differentiation process was calculated by scale package of R software using FPKM of different samples. Red is up-regulated and green is down-regulated (Permited by Kanehisa et al. 2017).

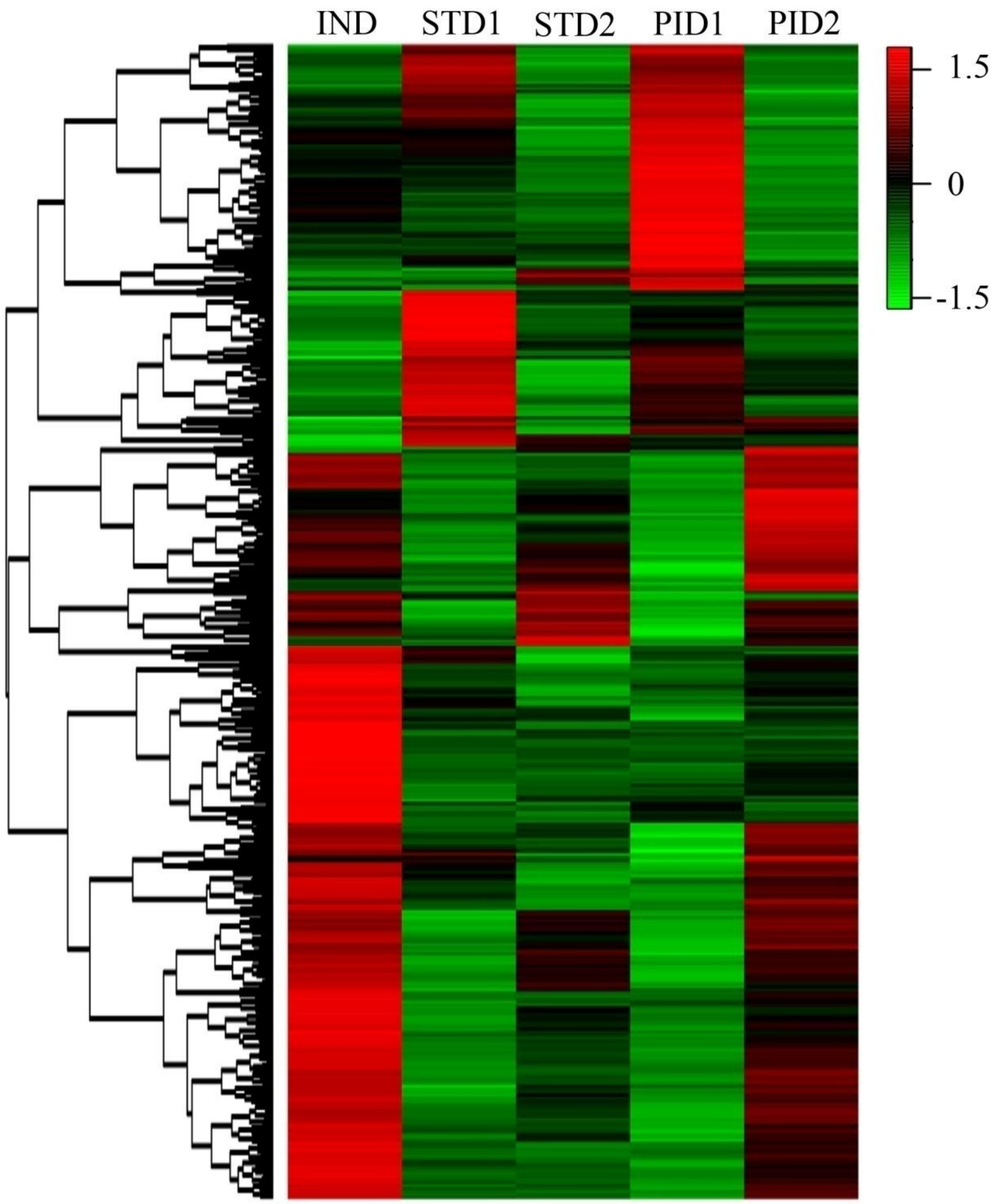

Supplementary Fig. S4 The expression profiles of male and female floral differentiation process related to DEGs in PID1*vs.*IND. Each column contained 395 DEGs detected in PID1*vs.*IND. Each row was the expression profiles in male and female floral differentiation process with the normalization method. Red is up-regulated and green is down-regulated.

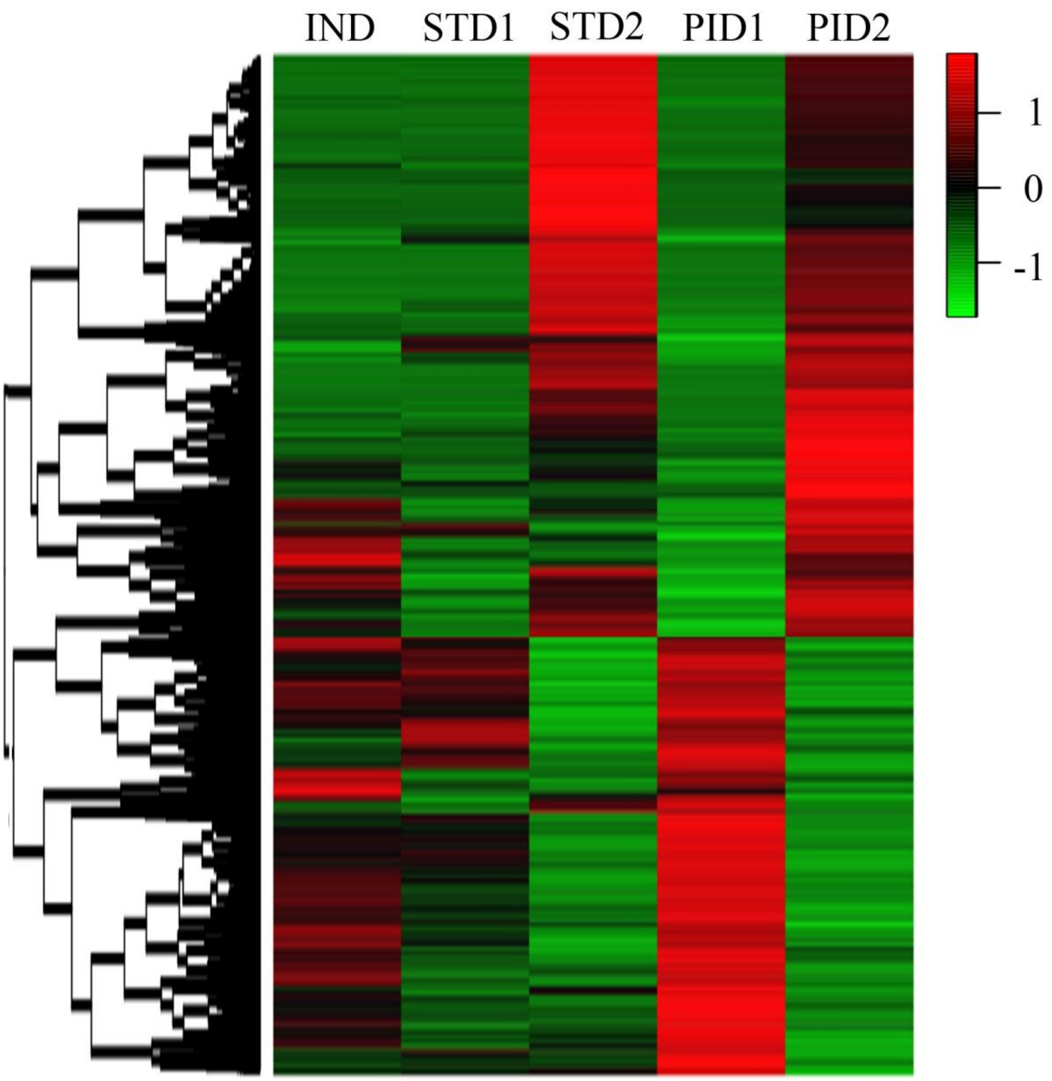

Supplementary Fig. S5 The expression profiles of male and female floral differentiation process related to DEGs in PID2*vs.*PD1. Each column contained 1,622 DEGs selected in PID2*vs.*PID1. Each row was the expression profiles in male and female floral differentiation process with the normalization method. Red is up-regulated and green is down-regulated.

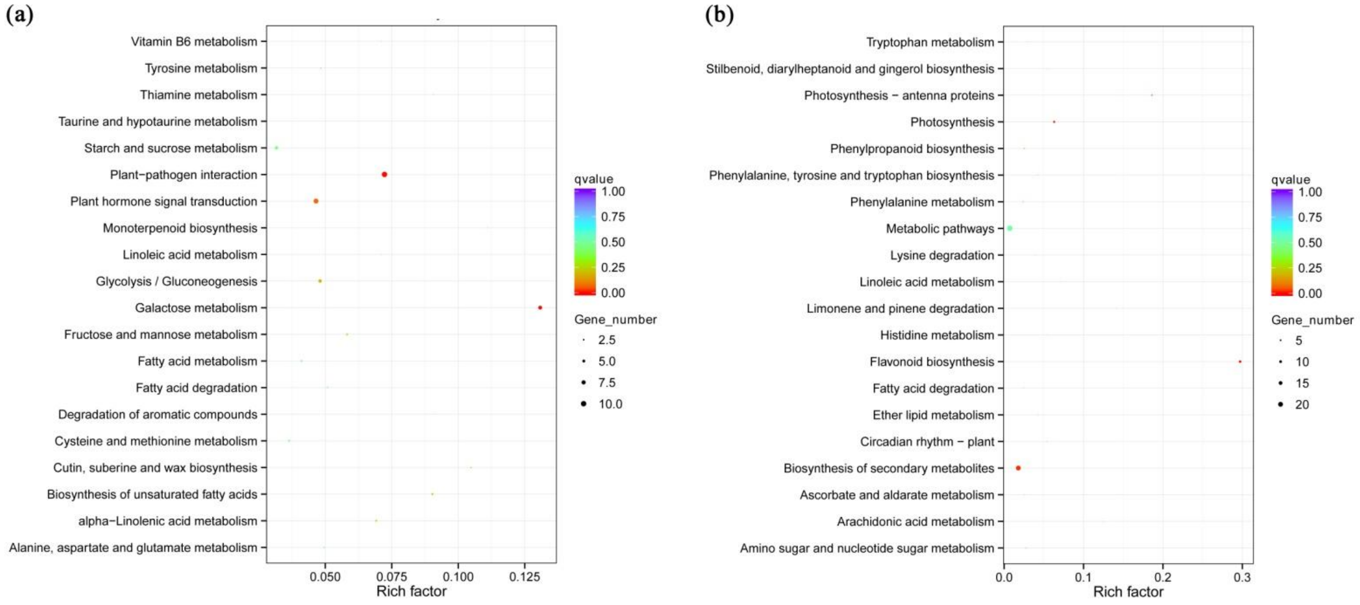

Supplementary Fig. S6 The KEGG enrichment pathways involved in PID1*vs.*STD1. The Rich factor indicated the percentages of DEGs belong to the corresponding pathway. The left y-axis represented the enrichment pathways. The sizes of bubble represent the number of DEGs in the corresponding pathway, and the colors of the bubble represent the enrichment Q value of the corresponding pathway. (a) KEGG enrichment pathways of the DEGs up-regulation in PID1*vs.*STD1. (b) KEGG enrichment pathways of the DEGs down-regulation in PID1*vs.*STD1.

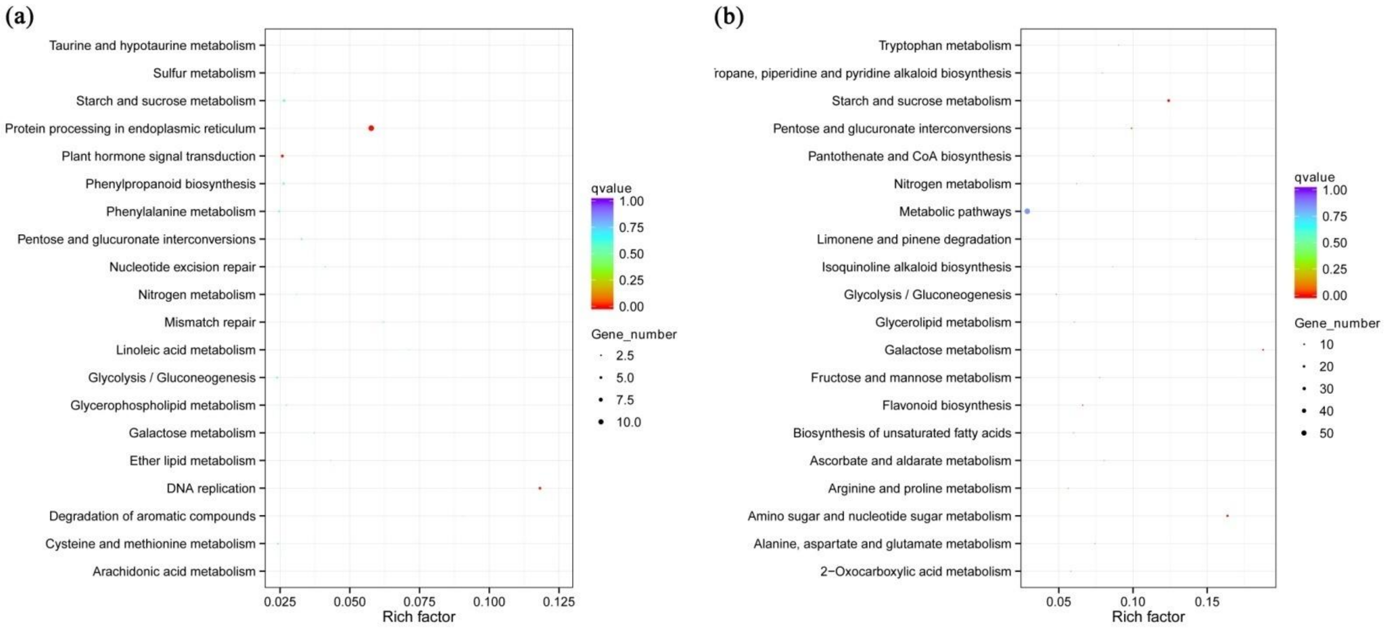

Supplementary Fig. S7 The KEGG enrichment pathways involved in PID2*vs.*STD2. The Rich factor indicated the percentages of DEGs belong to the corresponding pathway. The left y-axis represented the enrichment pathways. The sizes of bubble represent the number of DEGs in the corresponding pathway, and the colors of the bubble represent the enrichment Q value of the corresponding pathway. (a) KEGG enrichment pathways of the DEGs up-regulation in PID2*vs.*STD2. (b) KEGG enrichment pathways of the DEGs down-regulation in PID2*vs.*STD2.

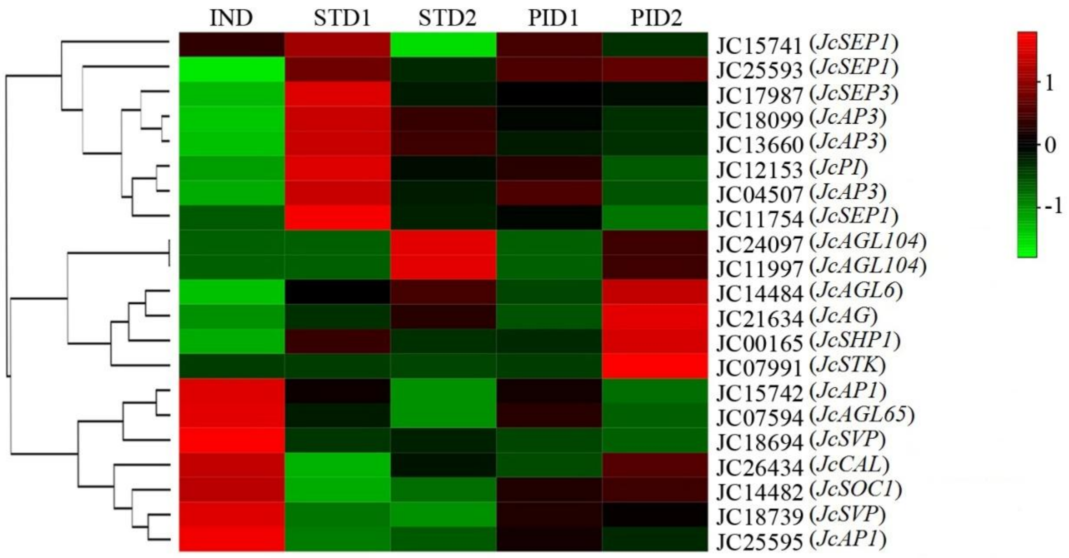

Supplementary Fig. S8 Heat map showing the relative expression level of *J. curcas* MADS-box DEGs detected in floral sex differentiation process based on RNA-seq data analysis. Z-score is calculated by scale package of R software using FPKM of different samples. Red is up-regulated and green is down-regulated.

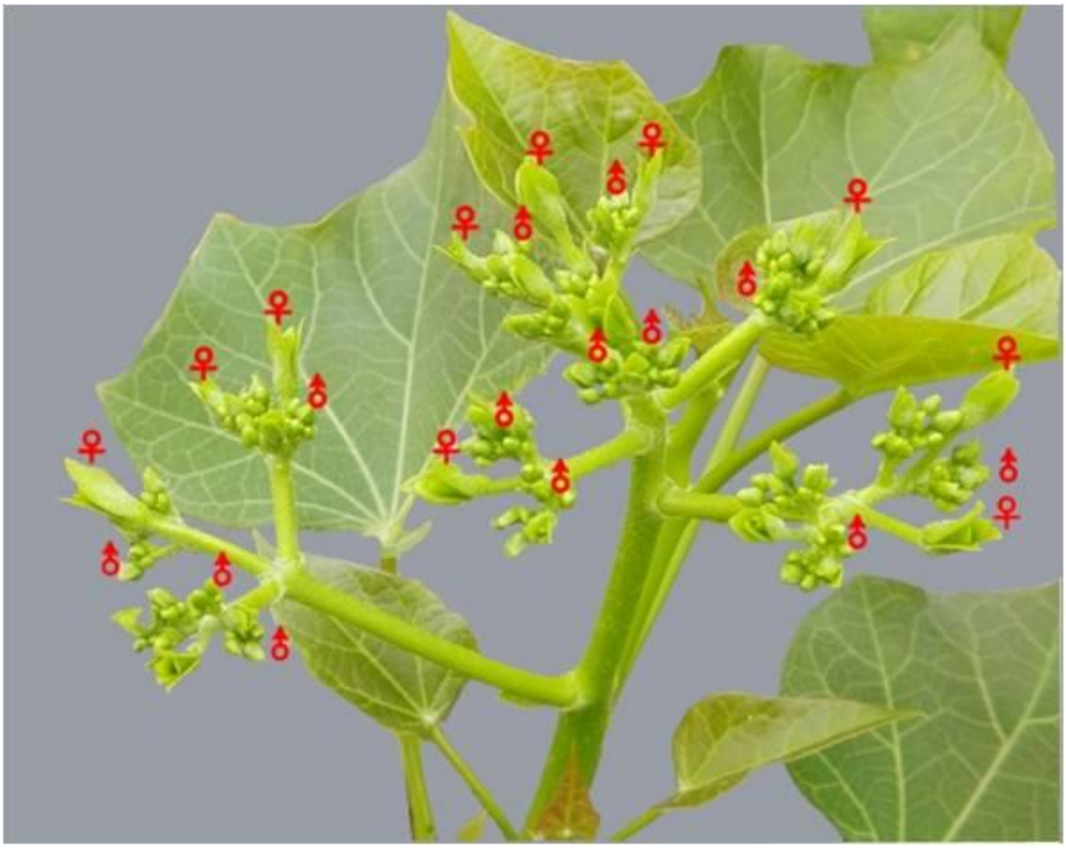

Supplementary Fig. S9 The configurations of male and female flower in *J. curcas* inflorescences. ♀represents the site of female flower; ♂represents the site of male flower.

Supplementary Table S1. The statistic results of floral differentiation stages

Stages	Collected number/No.	Number in the stage/No.	Percent/%
IND	10	10	100.00
STD1	30	30	100.00
STD2	30	30	100.00
PID1	30	28	93.33
PID2	30	30	100.00

Supplementary Table S2. The information about statistics of RNA-seq alignment

Sample name	Raw reads	Clean reads	Clean bases	Error rate (%)	Q20 (%)	Q30 (%)	GC content (%)
IND	53 085 642	49 704 048	7.46G	0.02	96.53	91.68	43.86
STD1	49 212 964	45 756 816	6.86G	0.02	96.48	91.71	43.81
STD2	57 115 000	52 253 148	7.84G	0.02	96.62	91.87	43.42
PID1	47 233 024	43 703 100	6.56G	0.02	96.29	91.29	43.88
PID2	51 253 614	47 638 618	7.15G	0.02	96.43	91.54	43.32

Supplementary Table S3. The information about the RNA-seq data mapped with the genome of *J.curcas*

Sample name	IND	STD1	STD2	PID1	PID2
Clean reads	49704048	45756816	52253148	43703100	47638618
Total mapped	40729019 (81.94%)	35566825 (77.73%)	41717169 (79.84%)	34311412 (78.51%)	38067823 (79.91%)
Multiple mapped	843901 (1.7%)	847052 (1.85%)	1170004 (2.24%)	764937 (1.75%)	945054 (1.98%)
Uniquely mapped	39885118 (80.25%)	34719773 (75.88%)	40547165 (77.6%)	33546475 (76.76%)	37122769 (77.93%)
Non-splice reads	26163714 (52.64%)	23040326 (50.35%)	25802865 (49.38%)	22000726 (50.34%)	23853337 (50.07%)
Splice reads	13721404 (27.61%)	11679447 (25.53%)	14744300 (28.22%)	11545749 (26.42%)	13269432 (27.85%)

Supplementary Table S4. The data of RNA-seq and qRT-PCR for validation of expression trend

Comparison	Gene ID	RNA-seq		qRT-PCR	
		Log2(Fold Change)	Fold Change	Log2($2^{-\Delta\Delta Ct}$)	$2^{-\Delta\Delta Ct}$
PID1*vs*.IND	JC01328	-1.16	0.45	-1.25	0.42±0.02
PID1*vs*.IND	JC09441	-1.32	0.40	-2.15	0.23±0.00
PID1*vs*.IND	JC13576	3.89	14.78	2.65	6.28±0.14
PID1*vs*.IND	JC18694	-1.48	0.36	-1.98	0.25±0.02
PID1*vs*.IND	JC23402	-1.12	0.46	-1.03	0.49±0.02
PID1*vs*.IND	JC25593	1.62	3.06	2.32	4.99±0.33
PID1*vs*.IND	JC25828	-1.89	0.27	-1.04	0.49±0.19
PID2*vs*.PID1	JC01392	-1.01	0.50	-1.21	0.43±0.01
PID2*vs*.PID1	JC02272	2.43	5.39	2.02	4.06±0.09
PID2*vs*.PID1	JC02934	-1.44	0.37	-1.96	0.26±0.01
PID2*vs*.PID1	JC04255	-2.29	0.20	-2.83	0.14±0.00
PID2*vs*.PID1	JC04805	-2.28	0.21	-2.68	0.16±0.01
PID2*vs*.PID1	JC05600	-1.19	0.44	-1.08	0.47±0.01
PID2*vs*.PID1	JC07511	-1.78	0.29	-2.46	0.18±0.00
PID2*vs*.PID1	JC07991	7.43	172.18	4.32	19.97±0.97
PID2*vs*.PID1	JC11537	-1.67	0.31	-1.04	0.49±0.05
PID2*vs*.PID1	JC12057	2.85	7.21	2.33	5.03±0.05
PID2*vs*.PID1	JC14204	-1.60	0.33	-2.11	0.23±0.01
PID2*vs*.PID1	JC14209	-1.28	0.41	-1.98	0.25±0.01
PID2*vs*.PID1	JC15742	-1.15	0.45	-1.05	0.48±0.01
PID2*vs*.PID1	JC16280	-1.87	0.27	-2.26	0.21±0.01
PID2*vs*.PID1	JC19628	4.14	17.59	3.89	14.83±0.65
PID2*vs*.PID1	JC21755	-1.78	0.29	-1.51	0.35±0.01
PID2*vs*.PID1	JC23114	-2.37	0.19	-1.69	0.31±0.01
PID2*vs*.PID1	JC23499	1.50	2.83	2.34	5.06±0.23
PID2*vs*.PID1	JC24672	-2.52	0.17	-2.16	0.22±0.01
PID2*vs*.PID1	JC25989	-1.95	0.26	-1.48	0.36±0.01
PID2*vs*.PID1	JC26194	-1.25	0.42	-1.79	0.29±0.00
PID2*vs*.STD2	JC06956	1.89	3.70	1.34	2.53±0.18
PID2*vs*.STD2	JC07165	1.11	2.16	2.01	4.03±0.23
PID2*vs*.STD2	JC13432	1.15	2.22	1.04	2.06±0.18
PID2*vs*.STD2	JC22124	2.40	5.27	2.11	4.32±0.32
STD1*vs*.IND	JC00165	3.31	9.89	2.01	4.03±0.15
STD1*vs*.IND	JC04507	1.81	3.50	2.25	4.76±0.74
STD1*vs*.IND	JC04785	9.20	588.83	5.46	44.02±3.92
STD1*vs*.IND	JC06233	1.88	3.68	1.34	2.53±0.05
STD1*vs*.IND	JC06610	1.24	2.36	2.17	4.50±0.67
STD1*vs*.IND	JC11710	1.25	2.37	1.47	2.77±0.38
STD1*vs*.IND	JC11754	1.19	2.28	1.68	3.20±0.15
STD1*vs*.IND	JC12153	3.57	11.87	2.65	6.28±0.14
STD1*vs*.IND	JC13660	4.01	16.11	3.48	11.16±0.49
STD1*vs*.IND	JC14482	-2.44	0.18	-1.46	0.36±0.01
STD1*vs*.IND	JC14484	1.38	2.60	1.88	3.68±0.51
STD1*vs*.IND	JC17975	-1.17	0.44	-1.38	0.38±0.02

STD1*vs*.IND	JC17987	2.92	7.55	3.65	12.55±0.70
STD1*vs*.IND	JC18099	3.58	11.96	2.98	7.89±0.84
STD1*vs*.IND	JC18282	2.18	4.53	1.57	2.97±0.13
STD1*vs*.IND	JC18739	-1.52	0.35	-2.01	0.25±0.02
STD1*vs*.IND	JC19526	-1.20	0.44	-1.46	0.36±0.03
STD1*vs*.IND	JC20688	1.30	2.46	1.77	3.41±0.11
STD1*vs*.IND	JC21298	1.98	3.94	1.44	2.71±0.04
STD1*vs*.IND	JC21634	2.50	5.64	1.87	3.66±0.30
STD1*vs*.IND	JC23029	6.27	77.09	5.43	43.11±4.20
STD1*vs*.IND	JC25510	5.35	40.67	8.21	296.11±11.61
STD1*vs*.IND	JC25595	-2.24	0.21	-2.01	0.25±0.01
STD1*vs*.IND	JC26434	-3.04	0.12	-2.11	0.23±0.01
STD2*vs*.STD1	JC07594	-3.43	0.09	-4.22	0.05±0.01
STD2*vs*.STD1	JC11997	4.58	23.97	3.97	15.67±5.58
STD2*vs*.STD1	JC15741	-1.30	0.41	-1.69	0.31±0.01
STD2*vs*.STD1	JC20786	2.20	4.59	2.03	4.08±0.73
STD2*vs*.STD1	JC24097	9.76	864.55	7.62	196.72±39.42
STD2*vs*.STD1	JC25229	1.41	2.66	1.58	2.99±0.47
STD2*vs*.STD1	JC05474	-5.77	0.02	-3.75	0.07±0.00
STD2*vs*.STD1	JC04645	-4.61	0.04	-2.68	0.16±0.00
STD2*vs*.STD1	JC16199	-4.54	0.04	-3.17	0.11±0.00
STD2*vs*.STD1	JC19019	-3.80	0.07	-3.22	0.11±0.00
STD2*vs*.STD1	JC14878	-3.47	0.09	-2.79	0.14±0.00
STD2*vs*.STD1	JC05749	8.30	314.28	5.61	48.94±0.20
STD2*vs*.STD1	JC26828	5.45	43.67	4.62	24.52±0.45
STD2*vs*.STD1	JC05254	6.14	70.55	5.37	41.25±1.76
STD2*vs*.STD1	JC25913	7.70	208.63	4.28	19.47±0.41
STD2*vs*.STD1	JC04730	-2.38	0.19	-1.87	0.11±0.00
STD2*vs*.STD1	JC21562	2.12	4.36	1.04	2.05±0.06
STD2*vs*.STD1	JC26283	2.15	4.44	2.57	5.95±0.13

Supplementary Table S5. The primers of qRT-PCR used for validation of expression trend

Gene ID	Forward Primers	Reverse Primers
JC01328	CTGCTCTAGAAGAATTTTGTGGGG	CCAGCCCTCTATCTCCAATAACAT
JC00165	ACTATCTGTGCTTTGTGATGCTGA	CGTAGTTTGGAGGATTCTTGTTGAT
JC01392	AGTGTCTGATACTGGCGAGTGATG	GACGCTGATATTGTCCGTGCTGT
JC02272	TGGAATGAAGGAGTTGTTGGTGCTA	ACCCGTGGACCATCCCAGTAAAT
JC02934	AAGGACAAGAATCACCTCCCAACT	CTTCACAAATGCTGCACTCAAAC
JC04255	TTAGCCAAAAGACAGTGGATGAAG	CCTATTCTGCTGAAACCCAAGTC
JC04507	AGTACCAGAAGACGCTTGGGATC	GGCAGTGTCCACATCTTGCTCTA
JC04645	GTGTAGCAATCAACAGCGAGCCA	CAACGCCCAGACCAAATAGAAGG
JC04730	AGGCTTTGAACGACAAGAACGG	CAGGAGGGAGTGGCACTTTAGG
JC04785	GGCCGAATAACAGCAATAGACAT	TGAGCAACAAATCCATACCATAAGG
JC04805	ATGAGTGCCTGAAATGTGGGATG	AACTTGGCAACTGCCTGATAGAG
JC05254	TGCTCGCAATGTGGAAAGTCG	CGGTGGTGGGTTCCTGACTG
JC05474	AATCCATTTCTCCCAAGACCTCA	TGGTAGTCAACCCTTCGATTTCA
JC05600	GATAATCACATGGACGCAAAACG	TAGACATCAGTAAATTCTCTTCTTA
JC05749	GTTTGCTTTGCTCATTACTCCCACT	TTCCGCTTTGACATCAGCTCCTA
JC06233	TTGGGTATTCATAAAGAGTATTCGG	GCATGACTCGATAAACATCTCCC
JC06610	TGTAGCGTTTTGGAAATCAGTAGG	ATAGAGGAGCCAGGAGGAAAGAC
JC06956	CATTGAAACTCCCATTAGCTATTGT	GTGTAGTCTTTCGCATACGCCTC
JC07165	CTGCGTTCATTGTCCATCCGTAA	CAGTCACTCCGTGAAGTCCACTC
JC07511	AAACTGCACAAATGACTATTTTCTA	CTTGACTGGAGCAGAAGGAAACC
JC07594	GAAGAGAGAGAACACAGGAGGGC	CCAGGTTTGACTGCTTCATCTTT
JC07991	ATTGTCAGTCCTTTGCGATGCT	GAGTCACCCATTAGATGCCTGTTT
JC09441	CTTCTAGCTTGGGTTCGCTGTCT	TCAAAGTCTCCAGGGTTTGTATGT
JC11537	GCAGTATCTTGGATTGGATGGAG	GCCAGGCATAGAGTAATCTGTCATT
JC11710	TGAAGGAGAATGCTGACGATGAG	TGTGCTTGAAGATGTATCAGTGCC
JC11754	GAACTCTCAGTTCTTTGCGATGC	GCCAGGTATGTTTGCTTCTAATGC
JC11997	AGAAACATCTTGTTGACACCTTGAC	TCTCCATTTGAACTACTTCCTTGC
JC12057	TGGCTAGACTTCCTGTGGTGGTA	AAACAATCCAACCCAACCTCCTA
JC12153	TCTGGTTATTTTCGCTAGTTCCG	GTCCATTTTCAAGGGCTTCCTC
JC13432	AACTTGAGATGGTAATGGGTACAGC	GAAGATCGTATTCGGAGTCGTCA
JC13576	CAGCAGCTAGAATCCAGTAGAATAAAG	CCACAGCCACCTAAGAACAACC
JC13660	GAAACAAAGACAGGAACCCAAAG	CCTTCATTCTCCACTAAACCGTATT
JC14204	ATGATAATGGCAGAATCTTCCTTG	TGGTTAGTTTTGGAGGTGTCTGTCT
JC14209	GTCCCGTTAAGGAGTCTGCCTAT	ATTCAGGGAAGCACGTTTGAGTA
JC14482	ATTACTGGGTGAAGGTCTGGGTT	CTTTTCTTGTAGTCGCTCAATCTGT
JC14484	GCTTTATGAATTTGGTAGTGCTGG	CAATGCTGTTATCTTGGGGAGTG
JC14878	ATCCAAGGGACAGCAAACAGAAG	CGACAAGGACGATAACGAAATCAG
JC15741	GCACTTGAATCATCATGGGAAGG	TCGCAAATTACTATCGCACAGC
JC15742	GTGATGCTGAGGTTGCTTTGATT	CTCTTGTGAATTAAGATCGTTTGCTAT
JC16199	TATGAATAGGGAACAGGTACTTGAC	CAAAGTCCTCTTCGTCTGGGTAA
JC16280	ATTACTACAGCGGTGGATTGCC	CCTACTTCAACATCAACAAGGTCAA
JC17975	CTCTGATAATGTCCCTGAAACGC	TCCTAAATCCTGTCCAACTACGG
JC17987	ACGAAGCAAATAAGACCCTGAAAC	CACTCCAATGGATGAAAGAAACC
JC18099	ATGGGTCGTGGAAAGATTGAGAT	GATATTGTCGATGTTGTAGGGCTAA
JC18282	ATGACTATTTCAACTCCCTTCCCC	AGAAGGAGAAGTTGTTGGAGTAGGC

JC18694	TAACGCTACCGCTAGACAAGTCA	ATGCTGGAGCTGCAATAATCAA
JC18739	AGAGAATGAGCGACTAAAGCAACA	GGTAACCCCAATCTAAGGAAAGTG
JC19019	ATGAGTGCTATGCTCTGCCTGAA	CAATGTAACCGTTGTCCAATCCC
JC19526	GAGTAAAGATAAAGCGGAGAAAGC	ACAGCCATGAGAAGATCATAACCA
JC19628	AAGAGATGTTCAAGTTTAGGCAAGA	TCACAAGGAATAGTAAGCCCCAT
JC20688	TGCTCTCACTTTCATCCTCCACA	GCTCAGTATTTCAATCGTGTCGC
JC20786	GCAACCCTGTTCTAGTCCCTCCT	TAAAAGTCTACGCATTCCCACATAC
JC21298	TCAGGCTCTATCTCAATAACGCAT	AGGGAAATCTCCAAAATCAGTAGG
JC21562	CTCGGCGTTCTTTAGACGGTTAC	CGCTGCCTCCTTAGCTTTGA
JC21634	GCGAAGATTGCTGAGAATGAAAG	GTGGATAATGATTGGTGGGTTGT
JC21755	CGGTGTTTAGAGGAAGGAGCAGA	GATAGACAGCGACGACGAGGAGT
JC22124	GGAACACATCAACACCACAAACC	CCACAGCATCTAAACGAGCAACA
JC23029	CATCACTGTGGTATTGCGATTTTAG	TGACTGTTAGATTGCTGCCCTTTT
JC23114	TTCCAAGTCTCGCTTTCCTAAGTT	CGGTTCCGTTGTAGAAGATCGT
JC23402	ATGGATTGTTTGGTTCAGTTGC	GTCTGGGTTGCTTTCATCTTGC
JC23499	CTGACCAAGAAACTGCTCCTCCT	ATTCTGACCCATTGTAGCCTTCC
JC24097	ATCTACCTATGACCCATCAACCG	TCCATGAGACGACATCGCATC
JC24672	ACTTGTAACCTTTTGAGTCAGTATCTGA	TATCAGGCAGTAGATTCAGTGTTGTT
JC25229	ATGTAACAGAGTCATGCAATGAAGC	GCAGGCAGGTTGAAATAGAAAGC
JC25510	GGAACCGTAAAGGACCAACAACT	TCTGAGCAAGTCTCCTCAATGTCT
JC25593	TGGGAAGGAGGTGAGCAAAGTA	GTTGTGGCTGTCATCTGGTCTG
JC25595	CTCCAAATGACACTGAATCACAGG	TCTCTTAGAGTCATGGAATCCAAATC
JC25828	TTAACTGTAGGAACAGCTACAACCC	AAATCACTTCTGGCGGGCTAC
JC25913	GCAATATGTGCGTTGGTTCGTT	ATGCCTTCCTCTGATTCTGGTAA
JC25989	GGGAACGATAAAATGGTGGGA	ATTCATCAGGTCTGTCAGGCTTAT
JC26194	TTGTGACAGCAGAGCCTGAGATT	TCGTCCTAAAGCAAGGCGAGTGA
JC26283	GCTTCGCTTATGTCTTCAGTTGC	TACCCTTTGCCTATACCGCCAC
JC26434	GAGAACCCAACAAACAGACAGGT	GAGCAAACAAACTTCAGCATCACA
JC26828	TCGTTGTTGTCGCAGTGGTTG	CTACTTGACGCCTCTTCCCTTTC
Jcactin	CTCCTCTCAACCCCAAAGCCAA	CACCAGAATCCAGCACGATACCA
JcGAPDH	TGAAGGACTGGAGAGGTGGAAGAGC	ATCAACAGTTGGAACACGGAAAGCC

Reference

Kanehisa, M., Furumichi, M., Tanabe, M., Sato, Y., & Morishima, K. KEGG: new perspectives on genomes, pathways, diseases and drugs. *Nucleic Acids Res*. **45**, D353-D361 (2017).
